# CONNECTOR, fitting and clustering of longitudinal data to reveal a new risk stratification system

**DOI:** 10.1093/bioinformatics/btad201

**Published:** 2023-04-20

**Authors:** Simone Pernice, Roberta Sirovich, Elena Grassi, Marco Viviani, Martina Ferri, Francesco Sassi, Luca Alessandrì, Dora Tortarolo, Raffaele A Calogero, Livio Trusolino, Andrea Bertotti, Marco Beccuti, Martina Olivero, Francesca Cordero

**Affiliations:** Department of Computer Science, University of Torino, Torino 10149, Italy; Department of Mathematics G. Peano, University of Torino, Torino 10123, Italy; Candiolo Cancer Institute, FPO IRCCS, Candiolo 10060, Italy; Department of Oncology, University of Torino, Torino 10060, Italy; Candiolo Cancer Institute, FPO IRCCS, Candiolo 10060, Italy; Department of Oncology, University of Torino, Torino 10060, Italy; Candiolo Cancer Institute, FPO IRCCS, Candiolo 10060, Italy; Department of Oncology, University of Torino, Torino 10060, Italy; Candiolo Cancer Institute, FPO IRCCS, Candiolo 10060, Italy; Department of Molecular Biotechnology and Health Sciences, University of Torino, Torino 10126, Italy; Department of Computer Science, University of Torino, Torino 10149, Italy; Department of Molecular Biotechnology and Health Sciences, University of Torino, Torino 10126, Italy; Candiolo Cancer Institute, FPO IRCCS, Candiolo 10060, Italy; Department of Oncology, University of Torino, Torino 10060, Italy; Candiolo Cancer Institute, FPO IRCCS, Candiolo 10060, Italy; Department of Oncology, University of Torino, Torino 10060, Italy; Department of Computer Science, University of Torino, Torino 10149, Italy; Candiolo Cancer Institute, FPO IRCCS, Candiolo 10060, Italy; Department of Oncology, University of Torino, Torino 10060, Italy; Department of Computer Science, University of Torino, Torino 10149, Italy

## Abstract

**Motivation:**

The transition from evaluating a single time point to examining the entire dynamic evolution of a system is possible only in the presence of the proper framework. The strong variability of dynamic evolution makes the definition of an explanatory procedure for data fitting and clustering challenging.

**Results:**

We developed CONNECTOR, a data-driven framework able to analyze and inspect longitudinal data in a straightforward and revealing way. When used to analyze tumor growth kinetics over time in 1599 patient-derived xenograft growth curves from ovarian and colorectal cancers, CONNECTOR allowed the aggregation of time-series data through an unsupervised approach in informative clusters. We give a new perspective of mechanism interpretation, specifically, we define novel model aggregations and we identify unanticipated molecular associations with response to clinically approved therapies.

**Availability and implementation:**

CONNECTOR is freely available under GNU GPL license at https://qbioturin.github.io/connector and https://doi.org/10.17504/protocols.io.8epv56e74g1b/v1.

## 1 Introduction

In the biological and medical fields, longitudinal data are valuable to explore the evolution of a given event and are expected to have higher predictive power than cross-sectional studies, in which variables are collected at a single time point across a sample population. The investigation of the evolution of a system delivers useful insights into (i) how the measurements change over time within samples; (ii) the time span of relevant events; and (iii) how time evolution is associated with clinical surveillance.

Longitudinal data come in many forms. However, their main characteristic is that they consist of portions of functions or curves, with quantities observed as they evolve through time. In regard to the modeling of temporal data, the state of the art of mathematical methodologies can be classified into two types of approaches: statistical models, in which no biological mechanisms are specified, and mechanistic models, in which all relations are specified (Kendall et al. 1999). The statistical approach makes few assumptions and provides no information about the underlying mechanisms of the system under study. Conversely, mechanistic models are more suited to yield biologically aware knowledge since they are based on a theoretical framework imposed by the modelers. In this context, we propose a workflow based on statistical methods for Functional Data Analysis (FDA; Ramsay and Silverman 2005; Ferraty and Vieu 2006). The fundamental aims of FDA are those of classical statistics for simple points in a general but finite dimension. However, the classical methods developed for finite dimension and independent observations cannot be directly applied to functions or curves. Indeed, being functional data, the sampled variables are strongly correlated and the problem becomes ill-conditioned in the context of multivariate linear models. By analyzing data that vary over time, FDA statistics provide the analytical ground to interpret longitudinal data.

Here, we present CONNECTOR, a data-driven and flexible framework to analyze and inspect longitudinal data through FDA, with the aim of offering a new perspective of mechanism interpretation. CONNECTOR provides several graphical visualizations, which support users throughout all the analytical steps and required parameter optimizations. CONNECTOR is able to fit and cluster temporal data with great flexibility and with an accuracy that highlights differences in the dynamics. To the best of our knowledge, no other available tools support users without advanced expertise in the statistical analysis of complex temporal data in such an informative and interpretative manner as our proposed software.

To illustrate the effectiveness of CONNECTOR, we leveraged cancer growth data. The collection of cancer growth data from pre-clinical models to investigate the mechanisms underlying cancer progression and to identify effective treatments for specific patients’ subsets has steadily increased in the last years. These data are retrieved from different types of biological material, i.e. cancer cell lines (Sharma et al. 2010), patient-derived xenografts (PDXs), and organoids (Hidalgo et al. 2014; Rizzo et al. 2021). In particular, PDXs are used to monitor tumor growth kinetics by evaluating the average percentages of tumor volume variations, and hence they rely on the repetition of measurements that are presumed independent. The analysis of these data is commonly limited to the analysis of variance or to cross-sectional evaluations using t-tests focused at a single time point, which investigate the punctual time effect among all study arms and lose longitudinal correlation. In some cases, a categorical system in which the average percentages are classified into clinical categories is implemented. Among the common approaches to statistically analyze tumor growth datasets, in Oberg et al. (2021), a linear mixed effects regression model was used to fit and compare the tumor area, after natural logarithm transformation. From a mechanistic perspective, a broad variety of mechanistic models can be used to fit growth data (Benzekry et al. 2014; Sarapata and De Pillis 2014; Kareva and Karev 2018). All these approaches do not consider data as a timeline of cancer progression but as independent points revolving around a knowledge-based reduction of the system’s convolution. The FDA-based methods from which CONNECTOR stems overcome this limitation.

We employed CONNECTOR to inspect the evolution of more than 1500 growth curves of PDXs derived from metastatic colorectal cancer (mCRC) and treated with the clinically approved anti-EGFR monoclonal antibody cetuximab. The clusters generated by CONNECTOR allow the identification of a subset of cetuximab-resistant tumors associated with previously unrecognized molecular and phenotypic features.

Nevertheless, we also report an extensive comparison of CONNECTOR versus classical growth models (Benzekry et al. 2014) and other functional clustering methods (Jacques and Preda 2014). Moreover, we distribute a Docker image to guarantee full reproducibility of the presented analyses and for ease of use.

## 2 Materials and methods

### 2.1 The CONNECTOR framework

CONNECTOR is a tool for the unsupervised analysis of longitudinal data, it can process any sample consisting of measurements collected sequentially over time. CONNECTOR is built on the model-based approach for clustering functional data presented in James and Sugar (2003), which is particularly effective when observations are sparse and irregularly spaced, as growth curves usually are.

**Figure 1.**
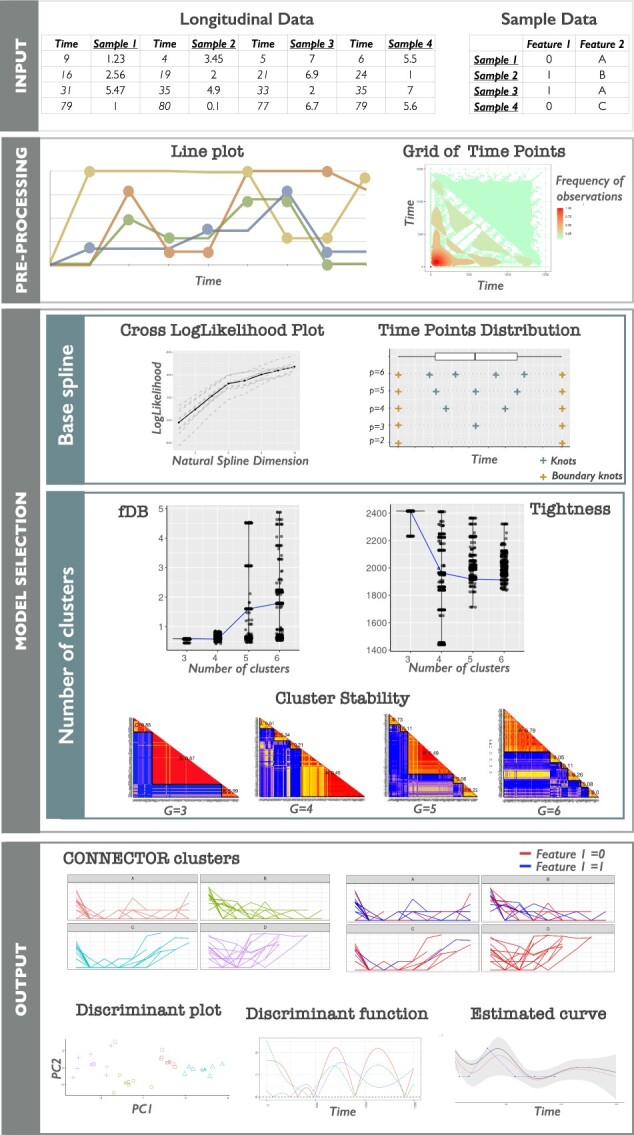
The framework pipeline of the CONNECTOR package. The four main stages of data processing are illustrated. The input data are the sampled curves, associated with annotation features. Data are pre-processed and curves are plotted. The heatmap of the full time grid is also provided. The model selection is supported with the cross-validated log-likelihoods and the positions of the knots for the choice of the dimension of the spline basis, and with violin plots for fDB (see Equation (5)) and the total tightness (see Equation (4)). Stability matrices are reported for the choice of the number of clusters. The output of the process is illustrated with the plots of the clustered curves.

Hereafter, an overview of the software is illustrated (Fig. 1), while the full description is reported in the Section 2. Any collection of observations recorded at several different time points, describing a system evolution, is accepted as a longitudinal dataset. These data, together with the description of each sample through a set of relevant features, are the input datasets of CONNECTOR.

The CONNECTOR framework is based on the following steps:

- The ‘pre-processing step’ consists of the visualization of the longitudinal data with a line plot and a heat map of the time points distribution to help in the inspection of the sparsity of the time points.
- The sampled curves are processed by CONNECTOR with a functional clustering algorithm based on a mixed-effect model. This step requires a ‘model selection phase’, in which several measures are computed to help the user properly set the two free parameters of the model: (i) the dimension of the spline basis vector, determined by the ‘cross-log-likelihood’ and the ‘knots distribution’; (ii) the number of clusters, determined by the ‘total tightness’, the ‘functional Davies–Bouldin (fDB) index’ jointly with the stability matrices.
- As ‘output’, the data dynamics are plotted in CONNECTOR clusters. The discriminant plot offers a visualization of the sample separation in the CONNECTOR clusters, projected on a plane. The discriminant function plot shows the discriminant power of each time point. The estimated curve, the confidence intervals, and the observations are reported for each sample.

### 2.2 Theoretical background

#### 2.2.1 Functional clustering model

The functional clustering method implemented in CONNECTOR is based on the functional clustering model presented in James and Sugar (2003). Let us denote as $g_{i}(t)$ the curve of the *i*th selected individual. In practice, we observe $g_{i}(t)$ with measurement errors and only at few discrete time points. Let $Y_{i}$ be the vector of observed values of $g_{i}(t)$ at times $t_{i_{1}},\ldots ,t_{i_{n_{i}}}$. Then we have where $g_{i}$ and $\varepsilon_{i}$ are the vector of true values and measurement errors at time grid, respectively. As there are only finite numbers of observations, individual curves are modeled using basis functions, in particular cubic splines. Let where s(t) is a p−dimensional spline basis vector and $\eta_{i}$ is a vector of spline coefficients. The $\eta_{i}$’s are treated with a random-effects model. In particular, the spline coefficients are modeled using a Gaussian distribution, where $z_{i}$ denotes the unknown cluster membership. Let *G* denote the true number of clusters. Cluster means are furthermore rewritten as where $\lambda_{0}$ and $\alpha_{k}$ are p− and h− dimensional vectors, Λ is a (*p*, *h*) matrix and h≤min(p,G−1). Thus, *h* represents the dimension of the mean space, allowing a further lower-dimensional representation of the curves with means in a restricted subspace (for h<G−1).


$$Y_{i}=g_{i}+\varepsilon_{i},$$

(1) $$g_{i}(t)=s{\left(t\right)}^{T}\eta_{i},$$

$$\eta_{i}=\mu_{z_{i}}+\gamma_{i},\;\gamma ∼N(0,\Gamma ),$$

$$\mu_{k}=\lambda_{0}+\Lambda \alpha_{k},$$

With this formulation, the functional clustering model can be written as where $S_{i}={\left(s(t_{i_{1}}),\ldots ,s(t_{i_{n_{i}}})\right)}^{T}$ is the spline basis matrix for the i−th curve. There are many possible forms for *R* and Γ. For now, we use $R=\sigma^{2}I$ and a common Γ for all clusters, as we are interested in sparse datasets, for which the smallest number of parameters is advisable.


(2) $$\begin{array}{c} Y_{i}=S_{i}\cdot (\lambda_{0}+\Lambda \alpha_{z_{i}}+\gamma_{i})+\varepsilon_{i},\;i=1,\ldots ,n,\\ \varepsilon_{i}∼N(0,R),\;\gamma_{i}∼N(0,\Gamma ),\;\;\; \end{array}$$

#### 2.2.2 Model selection

Two free parameters have to be properly chosen before fitting: the dimension of the spline basis *p* and the number of clusters to fit *G*.

The dimension of the spline basis can be chosen as the one corresponding to the largest cross-validated likelihood, as proposed in James (2000). CONNECTOR uses a 10-fold cross-validation, which involves splitting data into 10 roughly equal-sized parts, fitting the model to 9 parts and calculating the log-likelihood on the excluded part. Repeat 10 times and combine log-likelihoods. Notice that, the resulting plot of the mean tested likelihoods versus the dimension of the basis, should be treated as a guide rather than an absolute rule, keeping in mind that working with sparse data pushes to spare parameters. Moreover, as the position of the knots depends on their number, CONNECTOR returns a plot with this information as the parameter *p* varies.

The number of clusters *G* must be chosen. CONNECTOR provides two different plots to properly guide in one of the most difficult problems in cluster analysis. As in the finite-dimensional case, where data are points in place of curves, we need some proximity measures to validate and compare the results of a clustering procedure.

We chose to follow Ferraty and Vieu (2006) and rely on the parameterized family of semi-metrics between curves defined as where *f* and *g* are two curves and $f^{\left(q\right)}$ and $g^{\left(q\right)}$ are their *q*th derivatives. Note that for *q* = 0, Equation (3) is the distance induced by the classical $L^{2}-$norm. It turns out that *D_q_* can be reliably calculated in our setting where we are interested in proximity measures between each curve in a cluster and the center-curve of the cluster (tightness of the cluster), as well as proximity measures between each center-curve and the center-curves of different clusters (separateness of clusters). Hence, we may have *f* being the estimated *i*th curve and *g* being the estimated mean curve of cluster *k*, or *f* and *g* being both mean curves. In any case, *D_q_* can be calculated by taking advantage of the spline representation of the estimated curves and mean curves, see Equation (1). Indeed, the computation of successive derivatives, which is numerically sensitive, can be performed by differentiating their analytic form. Finally, the integral can be computed by numerical method. As the proper calculation of *D_q_* is a basic condition for the tools shown below, all details are illustrated in Supplementary Material S1.


(3) $$D_{q}(f,g)=\sqrt{\int {\left|f^{\left(q\right)}(s)-g^{\left(q\right)}(s)\right|}^{2}ds},\;q=0,1,2,$$

We define a first quantity to infer the appropriateness of the data partition, which we call *total tightness*. It is the dispersion measure given by where ${\hat{g}}_{i}$ is the estimated *i*–th curve given in Equation (S4) and ${\bar{g}}^{k}$ is the center of *k*-th cluster given in Equation (S3), see Supplementary Material S2. As the number of clusters increases, the total tightness decreases to zero, the value which is attained when the number of fitted clusters equals the number of sampled curves. In this limiting case, any *k*-th cluster mean curve coincides with an estimated curve and $D_{0}({\hat{g}}_{i},{\bar{g}}^{k})=0$ for any *i* and *k*. A proper number of clusters can be inferred as being large enough to let the total tightness drop down to relatively little values but small enough so that the total tightness does not decrease substantially. Hence, we look for the location of an “elbow” in the plot of the total tightness against the number of clusters.


(4) $$T=\sum_{k=1}^{G}\sum_{i=1}^{n}D_{0}({\hat{g}}_{i},{\bar{g}}^{k}),$$

We define a second index, which is a cluster separation measure. Following Davies and Bouldin (1979), we extend the well-known Davies–Bouldin (DB) index to the functional setting. Let us call the new index fDB. It is defined as follows where for each cluster *k* and k′ with *G_k_* the number of curves in the *k*-th cluster. The significance of Equation (5) remains unchanged with respect to the finite-dimensional case. It is the average of the similarity measures of each cluster with its most similar cluster. The “best” choice of clusters will be that which minimizes this average similarity. It should be noted that $M_{k\prime k}$ is the distance between centroids (mean-curves) of k′-th and *k*-th cluster. It serves to weight the sum $S_{k\prime }+S_{k}$, which is the total standard deviation of the clusters: k′-th and *k*-th clusters dispersion is measured compared to their relative distance.


(5) $${\text{fDB}}_{q}=\frac{1}{G}\sum_{k=1}^{G}\underset{k\prime \neq k}{\max}\left\{\frac{S_{k\prime }+S_{k}}{M_{k\prime k}}\right\},$$

$$S_{k}=\sqrt{\frac{1}{G_{k}}\sum_{i=1}^{G_{k}}D_{q}^{2}({\hat{g}}_{i},{\bar{g}}^{k})}\;\;\text{and}\;\;M_{k\prime k}=D_{q}({\bar{g}}^{k\prime },{\bar{g}}^{k}),$$

CONNECTOR returns the violin plots of both the total tightness and the fDB index for a given repetition of runs and for different choices of the number of clusters *G*. To support this decision, CONNECTOR also returns the consensus matrix for the most frequent clustering at each given number of clusters. The plot informs about the stability of the final clustering across different runs. Indeed, each cell of the matrix is colored proportionally to the frequency of the two corresponding curves belonging to the same cluster across different runs. Hence, the larger the frequencies (which correspond to warmer colors of the cells in the plot), the more stable is the final clustering. Motivated by its meaning, we refer to such a matrix as the stability matrix. The observation of the fDB violin plots, the total tightness violin plots together with the stability matrix allows the user to properly set the free parameter *G*, which represents the number of clusters to be fitted. Notice that the functional clustering method allows for a lower dimensional representation of the curves through the parameter *h*. This reduction is often needed as data could not be enough to estimate a large number of parameters of the functional clustering model. CONNECTOR optimizes the choice of the parameter *h* by returning the largest value for which the estimation of the parameters is successful with a reasonable frequency set by the user. Hence, the value of *h* is not chosen directly but returned by CONNECTOR.

#### 2.2.3 Functional clustering tools

Three tools to analyze the clustering are presented: the discriminant plot, the discriminant function, and the estimation of the entire curve for each single subject.

The ‘discriminant plot’ is a low-dimensional plot of the curves dataset. It helps to visualize the clusters, as each curve is projected in a low-dimensional space so that it can be plotted as points. In particular, each curve is represented by its projection onto the *h*-dimensional space spanned by the means $\mu_{k}$.

The ‘discriminant functions’ are plots of the weights $\Lambda^{T}S^{T}\Sigma^{-1}$, versus time, to apply to each dimension for determining cluster membership. The term SΛ is a measure of average separation between clusters and Σ is a measure of their variability. There will be *h* discriminant functions and each curve shows the times with higher discriminatory power, which are the times corresponding to largest absolute (positive or negative) values on the y-axis. The functional clustering procedure predicts unobserved portions of the true curves for each subject. The ‘estimated curves’ are returned by CONNECTOR and plotted with confidence intervals as well. Supplementary Material S3 is reported an exhaustive review on the performance of different procedures with respect to the results obtained by CONNECTOR.

### 2.3 Details on the computational methods and further experiments

In Supplementary Material S1, a complete overview of the CONNECTOR framework can be found, as well as the computational details, see Supplementary Material S5 and S6. An evaluation of CONNECTOR performances is reported in Supplementary Material S7 and a comparison with different functional clustering method is presented in Supplementary Material S8. All methods and materials referred to validating xenograft experiments are included in Supplementary Materials S2.

## 3 Results

### 3.1 Application of CONNECTOR to PDX curves of metastatic colorectal cancer

To show the full potential of CONNECTOR in a complex use case, we analyzed a large dataset of tumor growth curves of PDXs from mice treated with cetuximab, an anti-EGFR antibody that is approved for clinical use in patients with RAS/RAF wild-type mCRC. Results from these xenotrials were obtained from a continuously expanding collection of ∼400 mCRC PDXs, part of which had been used in previous studies (Bertotti et al. 2011, 2015; Zanella et al. 2015; Isella et al. 2017; Lupo et al. 2020). Tumor volumes were measured weekly after tumor implantation and over the course of treatment. In previous projects, we categorized PDX response to cetuximab based on parameters that are loosely inspired by the RECIST clinical criteria (Bertotti et al. 2011; Ko et al. 2021). This classification includes three classes, based on the average tumor volume variation at the endpoint compared with tumor volume at baseline (the day before treatment initiation): partial responses (PR) are defined as tumors that regress by 50% or more during treatment; progressive diseases (PD) are defined as tumors that increase their volume by 35% or more, despite treatment; tumor volume changes above PR or below PD thresholds are defined as stable diseases (SD). To allow a direct comparison of CONNECTOR’s clustering results with our historical annotation, we decided to analyze, for each tumor, the available measurements between the day before treatment initiation and the following 3 weeks. The selected dataset was extracted from the laboratory LIMS (Baralis et al. 2012) and comprises measurements from 1563 individual mice, collected from 2012 to 2020 and representing 173 original engraftments from parental tumors in patients.

The PDX curves were analyzed by CONNECTOR to achieve a widespread overview of the distribution of the individual parental tumor after drug administration. When running CONNECTOR, based on the results from the *model selection* phase, the optimal value of the base spline was 4. To perform a thorough analysis, we evaluated fDB and tightness for a broad range of cluster numbers, starting with three, which corresponds to the number of clinical response classes. We chose to compare the results obtained with 3, 4, and 5 clusters, as the fDB index worsened for larger values, see Supplementary Fig. S1A.

**Figure 2.**
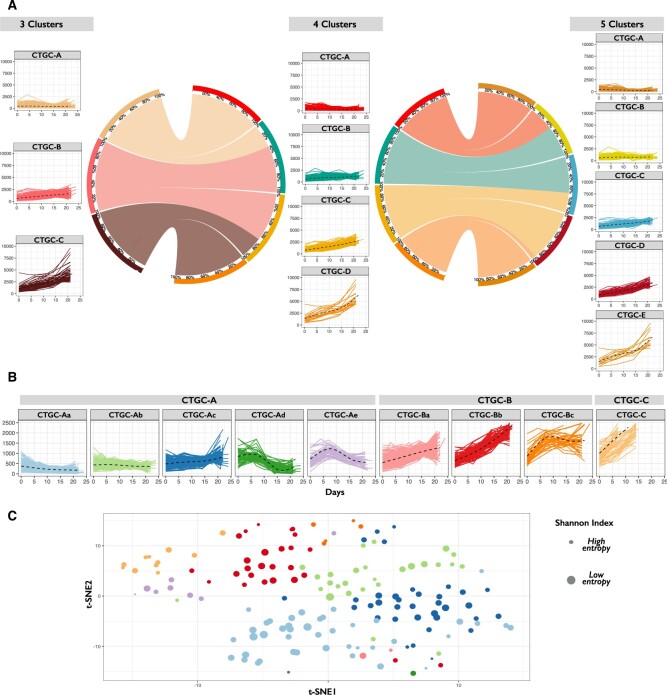
CONNECTOR results. (A) CONNECTOR tumor growth classes (CTGCs) for three, four, and five clusters. The circos plots show the repositioning of the curves as the number of CTGCs changes. (B) CONNECTOR tumor growth classes with 2-fold clustering. The nine boxes result from a first run with a number of clusters equal to three followed by second runs on the CTGC-A (with five sub-classes) and on the CTGC-B (with three sub-classes). To make more appreciable the differences among the clusters the y-axis reaches the maximum value of 2500 mm^3^. (C) t-SNE visualization of the CTGCs induced on the parental tumors. Each dot corresponds to a parental tumor. The color of the dots matches the color of the assigned CTGC, see Panel B. The dimension of the dots is inversely proportional to the Shannon Index calculated on the distribution of the curves of the same parental tumor across CTGCs (large dots—small entropy).

We studied how the PDX growth curves are distributed ‘intra-parameter setting’, among the number of CONNECTOR tumor growth classes (CTGCs) selected for each run, and ‘inter-parameter setting’, exploring how the curves move among the CTGCs along the three runs (Fig. 2A). We observed that by increasing the number of CTGCs, the curves that were separated were mainly those characterized by a sudden volume increase. Based on such analysis, the dataset was optimally described by three primary classes, namely CTGC-A, -B, and -C, leading to an average stability matrix of 1. We then assessed whether a higher resolution could be achieved by segregating the larger CTGCs into subclasses. We thus processed again, as independent datasets, the CTGCs composed by ˃200 curves (namely CTGC-A and -B, see Supplementary Fig. S1B and C, respectively). Through this, we obtained a final number of 9 CTGCs—where CTGC-A and B are further split into Aa, Ab, Ac, Ad, Ae, and Ba, Bb, and Bc—which are represented in Fig. 2B. See Supplementary Material S3 for a detailed description of this step of the analysis.

We assigned each parental tumor to a specific CTGC by means of a Naïve Bayesian classifier, using the membership probabilities returned by CONNECTOR for each PDX curve. By calculating the Shannon index, representing the consistency of such assignment of the parental tumor to a CTGC from the CTGCs of the PDX curves, we observed that in most cases there is high agreement (mean = 1.54, s.d. = 0.52). For the sake of clarity, an additional graphical visualization, based on the t-distributed stochastic neighbor embedding (t-SNE) is proposed (Fig. 2C). Observing the scatter plot, it is appreciable that distinctly isolated CTGCs, by focusing on the color and the size of the dots, suggest their coherence. See Supplementary Material S4 for the computational details.

### 3.2 CONNECTOR clusters reveal a subgroup of non-responder tumors that express high levels of stratified epithelium keratins

**Figure 3.**
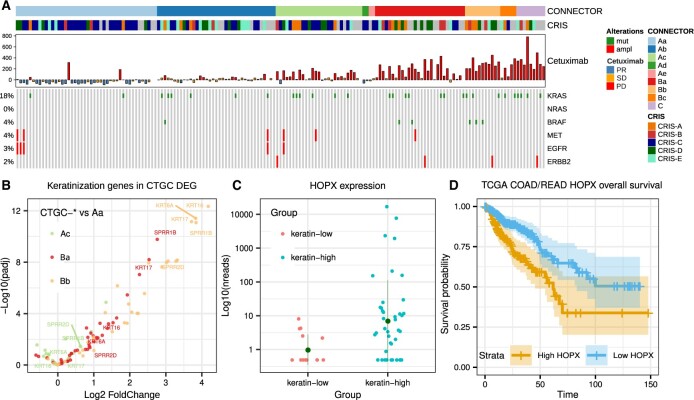
CONNECTOR clusters molecular annotation and transcriptomic analyses. (A) Molecular and phenotypic characterization of the CONNECTOR clustered mCRC xenografts: each sample was annotated according to CRIS subtype, response to cetuximab and somatic alteration known to determine cetuximab resistance or sensitivity. (B) Differential expression of genes in the “keratinization” GO in CTGCs: volcano plot showing the magnitude of expression differential (*x*-axis, Log2 FoldChange) and significance (*y*-axis, −Log10 adjusted *P* value) of “keratinization” genes when comparing CTGCs enriched in PD versus Aa. Only comparisons involving CTGCs enriched in PD with at least nine total samples are reported for clarity. The top five upregulated genes in Bb are labeled (SPRR a family of proteins induced during the differentiation of keratinocytes). (C) HOPX expression in keratin-high and keratin-low samples: the *y*-axis shows DESeq2 corrected counts. (D) Survival analysis of TCGA COAD-READ (colon and rectum adenocarcinoma) patients stratified by HOPX expression levels: survival time is in months, 134 (high) and 237 (low) patients. Log-rank *P*-value $8.6\times 10^{-3}$.

Different CTGCs were evaluated with respect to our three-class cetuximab response annotation and the molecular characteristics of the classified tumors (Fig. 3A).

As expected, CTGCs were associated with RECIST-like response classes. Indeed, PR tumors were enriched in CTGC-Aa (Fisher *P*-value $2.4\times 10^{-13}$), and SD tumors were enriched in CTGC-Ab (Fisher p-value $8.0\times 10^{-8}$). Notably, CONNECTOR segregated PD tumors in multiple clusters (p values: CTGC-Ac $5.5\times 10^{-2}$; CTGC-Ba $1.9\times 10^{-4}$; CTGC-Bb $7.5\times 10^{-5}$; CTGC-Bc $1.7\times 10^{-1}$; CTGC-C $4.6\times 10^{-4}$), indicating that CONNECTOR can distinguish different growth patterns within the PD standard response category.

Each CTGC is associated with the transcriptional subtypes identified by a PDX-based cancer cell-intrinsic (CRIS) classifier (Isella et al. 2017). In detail, the classification is based on the expression levels of different sets of subtype-specific genes via the nearest template prediction algorithm (Hoshida 2010). This association showed interactions between the growth patterns detected by CONNECTOR and specific biological traits.

In line with the observation that cetuximab-responsive tumors are enriched in the CRIS-C subtype (Isella et al. 2017), CRIS-C tumors were associated with the CTGC-Aa cluster. However, the enrichment of CRIS-C tumors within CTGC-Aa (Odds Ratio CRIS C/Aa 4.63) was stronger than that observed when considering all responders (Odds Ratio CRIS-C/PR 3.38). This suggests that CONNECTOR is able to recognize the specific subset of responders sustained by the CRIS-C phenotype.

The finding that CTGCs accurately captured specific molecular features prompted us to investigate whether the diversification of cetuximab-resistant cases in multiple CTGCs could relate to different biological substrates of resistance. Even if, as expected, all variants known to cause resistance to cetuximab in CRC (Bertotti et al. 2015) were depleted from CTGC-Aa (Fisher *P* value $1.3\times 10^{-2}$) and, albeit to a non-significant extent, from CTGC-Ab (Fisher *P* value 0.22), we did not observe an enrichment for specific resistance mutations in any of the PD-associated CTGCs, suggesting that the tumor growth patterns recognized by CONNECTOR in non-responders were not driven by specific resistance genotypes.

To further explore the functional characteristics of the different PD-enriched CTGCs, we mined a set of RNA-seq data that was generated in the context of an independent study performed on the same cohort (Perron et al. in preparation). Analysis of differentially expressed genes (DEGs) between all PD-enriched CTGCs and CTGC-Aa, used as a common reference, uncovered a very specific Gene Ontology (GO) enrichment related to stratified epithelial differentiation and keratinization, which was guided by keratin-encoding genes significantly upregulated in CTGC-Ba and CTGC-Bb, but not in CTGC-Ac (Fig. 3B;Supplementary Table S1; Supplementary Fig. S2E–F). This was not simply driven by the enrichment of non-responder tumors in CTGC-Ba and CTGC-Bb. Indeed, the same functional enrichments for CTGC-Ba and CTGC-Bb tumors versus CTGC-Ac tumors were observed also when limiting the analysis to PD tumors only (see Supplementary Table S2 and Supplementary Fig. S2A–C). Hence, CTGC unveiled two distinct phenotypes associated with cetuximab resistance, one of which is characterized by markers of keratinized stratified epithelia.

To validate this observation, we stratified the full cohort of cetuximab-resistant PDXs for which RNAseq data were available (*n* = 140) based on the expression of genes that were robustly upregulated in CTGC-Ba or CTGC-Bb and associated to “keratinization” according to GO annotations (details in Supplementary Material S3) and we identified 42 keratin-high samples and 15 keratin-low samples. H&E sections of four keratin-low and four keratin-high PDXs were subjected to blinded histopathological evaluation, which confirmed the existence of two clearly distinct subpopulations (see Supplementary Fig. S3). The first included tumors displaying histological features that resembled the glandular organization typical of the large intestine. Tumors belonging to the second group were less differentiated, with round or columnar pleomorphic cells that formed solid fields and rare luminal spaces. Intriguingly, in three out of four tumors of the latter group, we observed a variable degree of squamous differentiation. Indeed, after unblinding, three of the selected keratin-high tumors were found to belong to the second group, confirming that CTGC-B gene expression traits were likely associated with the acquisition of histological traits typical of squamous epithelia.

DEG analyses comparing keratin-high and keratin-low tumors confirmed the GO enrichments previously observed in CTGC-B tumors (see Supplementary Table S3 and Supplementary Fig. S4). Furthermore, novel associations to terms related to cell motility, wound healing, and angiogenesis emerged. This may indicate that the keratin-high subset is endowed with more aggressive and invasive properties with respect to keratin-low tumors. Interestingly, we found a robust enrichment for CRIS-B (fisher *P* value $2.09\times 10^{-6}$) among keratin-high tumors. CRIS-B was previously reported as an aggressive transcriptional subtype associated with poor prognosis (Isella et al. 2017) and undefined etiology. Our data may indicate that the CRIS-B phenotype is sustained by an aberrant differentiation pattern toward epithelial cornification.

The keratinocytic transdifferentiation of CTGC-B tumors may be driven by the activation of a stemness-related pathway sustained by the transcription factor HOPX, which is indeed upregulated in our keratin-high subgroup (Log-fold change 3.1, adjusted *P* value $4.23\times 10^{-6}$, Fig. 3C). Although the role of HOPX in CRC is controversial (Yamashita et al. 2013; Dmitrieva-Posocco et al. 2022), the assumption that its activation could contribute to the progression of a subset of aggressive tumors is in agreement with the observation that high expression of HOPX is significantly associated with bad prognosis in CRC (overall survival Log Rank *P* value $8.6\times 10^{-3}$ in the TCGA colorectal cohort (Fig. 3D;Liu and Zhang 2020).

## 4 Discussion

The main goal of this study was to develop a tool for the exploration of longitudinal data with an unsupervised approach. Hence, we worked on a tool to aggregate curves into classes, which can suggest directions and hypotheses for the in-depth examination of different datasets.

We reviewed the current literature on FDA and chose to build CONNECTOR based on the functional clustering method proposed by James and Sugar (2003). Indeed, depending on the sampling strategy (high frequency or sparse, regular, or irregular), different methods have been proven efficient for the clustering task. We were interested in curves that are observed at sparse and irregular times, as it usually occurs when data collection is laborious and time limits are constrained by ethical protocols. On these premises, and considering the results illustrated in Jacques and Preda (2014), we compared the best reported methods on sparse and irregularly sampled curves. The tests indicated that James and Sugar (2003) was the best performing method. The method has been studied to solve the main problems that arise when clustering sparse and irregularly sampled functional data: large numbers of missing observations on the discretized time grid (sparsity) and different covariances of the coefficients (curves are measured at different time points). Indeed, the method uses basis functions to project each curve onto a finite-dimensional space and considers a random-effect model for the coefficients. This allows to take advantage of all the sampled curves. The model is extremely flexible and computes estimates, confidence intervals, and prediction intervals for individual curves.

The performances of the functional clustering model are strongly dependent on the choice of several free parameters. CONNECTOR includes a toolset to choose each free parameter appropriately. With this aim, two new indexes have been introduced—fDB index and total tightness. The two indexes, together with the cross-log-likelihood plot, the visualization of the positions of the time knots and the stability matrix of the final clustering, provide the user with all the information needed to choose suitable values of the free parameter.

Oberg et al. (2021) have recently proposed a linear mixed-effects regression model for the analysis of PDX data obtained for repeated measurements. The model was able to fit different treatment designs and randomization schemes. However, their model is limited in the prediction of the trajectories since only monomials up to cubic degree are considered to describe time dependency, after log transformation. Conversely, the core of the CONNECTOR package is a general mixed-effects model, where both fixed-effects term and random-effects term are considered on a cubic spline basis, which makes the model as flexible as needed.

The versatility of CONNECTOR was also assessed by analyzing the spontaneous growth patterns of 21 PDX lines propagated from a single chemotherapy-naive high-grade serous epithelial ovarian cancer tumor sample. We observed uneven growth rates in PDX lines derived from the same original tumor. This is consistent with the notion that ovarian cancers show a high degree of intratumor heterogeneity. These results are reported in the Supplementary Material.

CONNECTOR allowed us to perform an in-depth study of the growth dynamics of a vast cohort of mCRC PDXs treated with cetuximab, with validation and discovery aspects. First, the reliability of the unsupervised and data-driven identification of CTGCs is supported by the observation that clusters were properly enriched in the three classes by which cetuximab response was previously annotated. Moreover, our analysis adds new insights into how cetuximab-resistant tumors can be stratified according to their molecular diversification. Specifically, CTGC-based categorization allowed us to identify a subset of cetuximab-resistant tumors (CTGC-B) with transcriptional and morphological features of metaplastic differentiation toward cornified epithelia (keratin-high tumors). CTGC-B tumors were also enriched for the CRIS-B transcriptional subtype, which was reported as a poor-prognosis tumor subgroup, composed of highly invasive tumors that are resistant to currently available therapeutic options (Isella et al. 2017).

Through CONNECTOR, we identified a previously unnoticed characteristic of such tumors, which may—at least partially—explain their aggressiveness and, at the same time, their keratinized phenotype. A literature search suggested the transcription factor HOPX as a potential regulator of the transdifferentiation process experienced by the keratin-high subgroup of cetuximab-resistant tumors (Takeda et al. 2013). HOPX is involved in the modulation of stem cell renewal both in the intestine and in the epidermis (Mariotto et al. 2016). In the latter, HOPX is responsible for the production of keratin 6-positive cells, which contribute to the post-injury regeneration of cornified epithelia by entering proliferation at the wound edge (Moll et al. 2008).

HOPX proved to be strongly upregulated in keratin-high, CTGC-B CRC tumors, and we provide evidence that HOPX expression correlates with poor prognosis in CRC patients. On this ground, we speculate that the same program that regulates the regeneration of keratinized epithelia may be aberrantly activated in CRC by HOPX overexpression, thus committing cancer cells toward an aggressive phenotype with traits of epithelial cornification. In this regard, it is worth noting that high expression of keratin 80, one of the markers of the keratin-high subgroup, has been associated with poor prognosis in CRC and with CRIS-B specific phenotypic traits, such as epithelial to mesenchymal transition and cell invasion (Li et al. 2018). This finding may have substantial implications. Indeed, if functionally validated, the notion that HOPX is a driver for the maintenance of keratin-high tumors with keratinocytic metaplastic differentiation may pave the way for the development of new therapeutic approaches aimed at interfering with HOPX function. We also note that keratinization did not stand out as a predominant characteristic of CRIS-B in the original study (Isella et al. 2017), possibly because the phenotype is specifically associated with the subpopulation of CRIS-B tumors that are resistant to anti-EGFR therapy. This makes CONNECTOR a versatile tool for discerning relevant trends from longitudinal data, which are harder to detect using more classical sources of molecular and phenotypic annotation.

Preclinical cohort studies involving the collection of longitudinal high-dimensional data are being increasingly conducted to evaluate drug activities and to explore disease evolution, and results from such efforts show potential to be translated into experimental trials and clinical practice. Disease monitoring over time is also an already well-established method that is routinely incorporated in several clinical trials. The CONNECTOR framework was designed and implemented as a user-friendly tool to streamline gathering this kind of information, while providing hints to increase interpretability and molecular accuracy.

## Supplementary Material

btad201_Supplementary_Data

## Data Availability

RNAseq data for mCRC xenografts are available here: https://www.ncbi.nlm.nih.gov/geo/query/acc.cgi?acc=GSE204805 Underlying raw data are available on EGA under restricted access: https://ega-archive.org/studies/EGAS00001006492.
