# Supplementary material for: CONNECTOR, fitting and clustering of longitudinal data to reveal a new risk stratification system
Source: Bioinformatics. 2023 Apr 20;39(5):btad201. doi: 10.1093/bioinformatics/btad201 (PMC10159654; doi:10.1093/bioinformatics/btad201)
Supplement: btad201_Supplementary_Data [file btad201_supplementary_data.pdf]

Supplementary Material

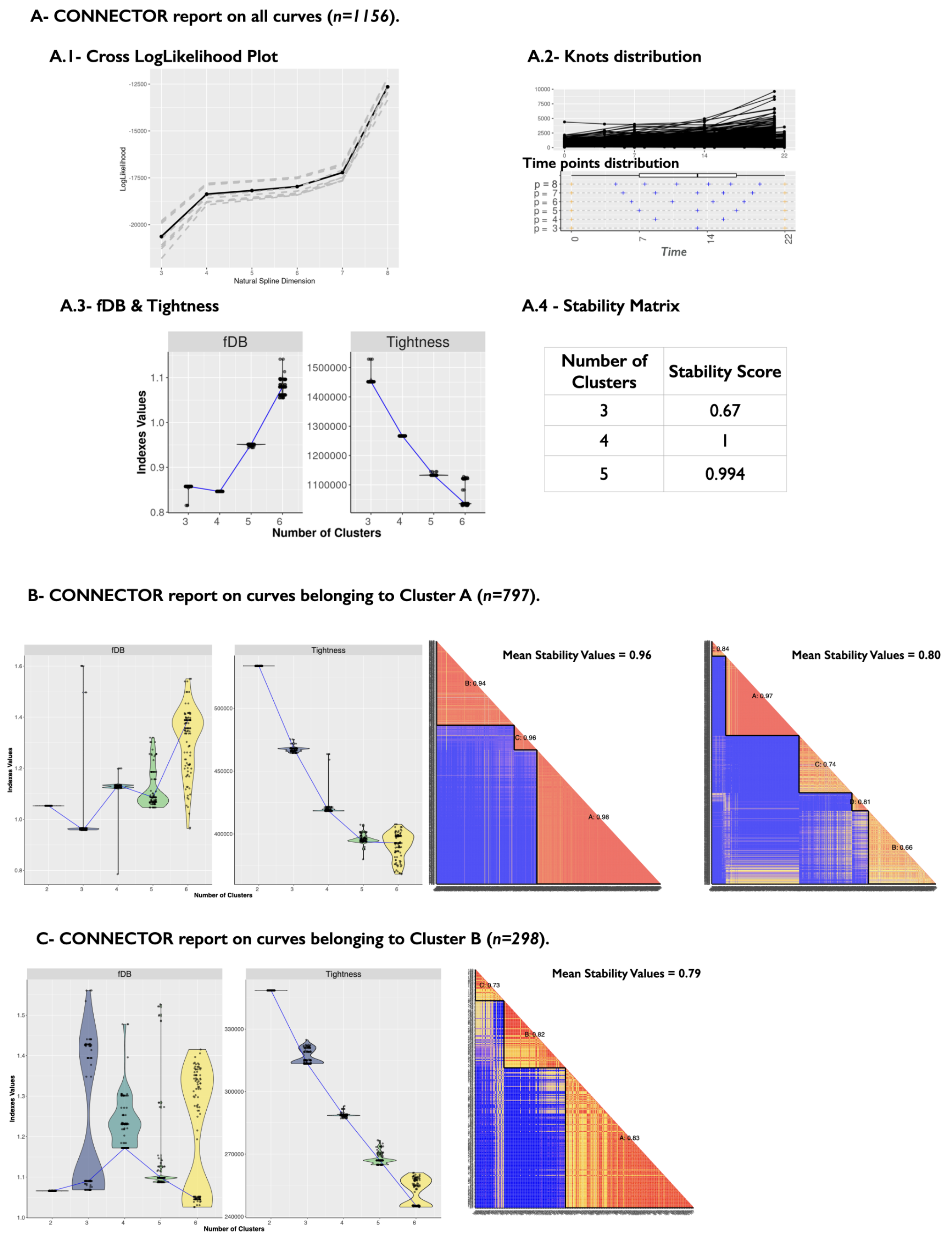

Fig. S1: **A** Cross LogLikelihood plot, fDB, and tightness obtained from the CONNECTOR run on all 1536 individual mice. The optimal set of parameters selected is p=4, h=2, and G=3. **B** fDB, tightness grid, and stability matrix obtained on cluster A. The optimal set of parameters selected is p=4, h=3, and G=5. **C** fDB, tightness grid, and stability matrix obtained on cluster B. The optimal set of parameters selected is p=4, h=3, and G=3.

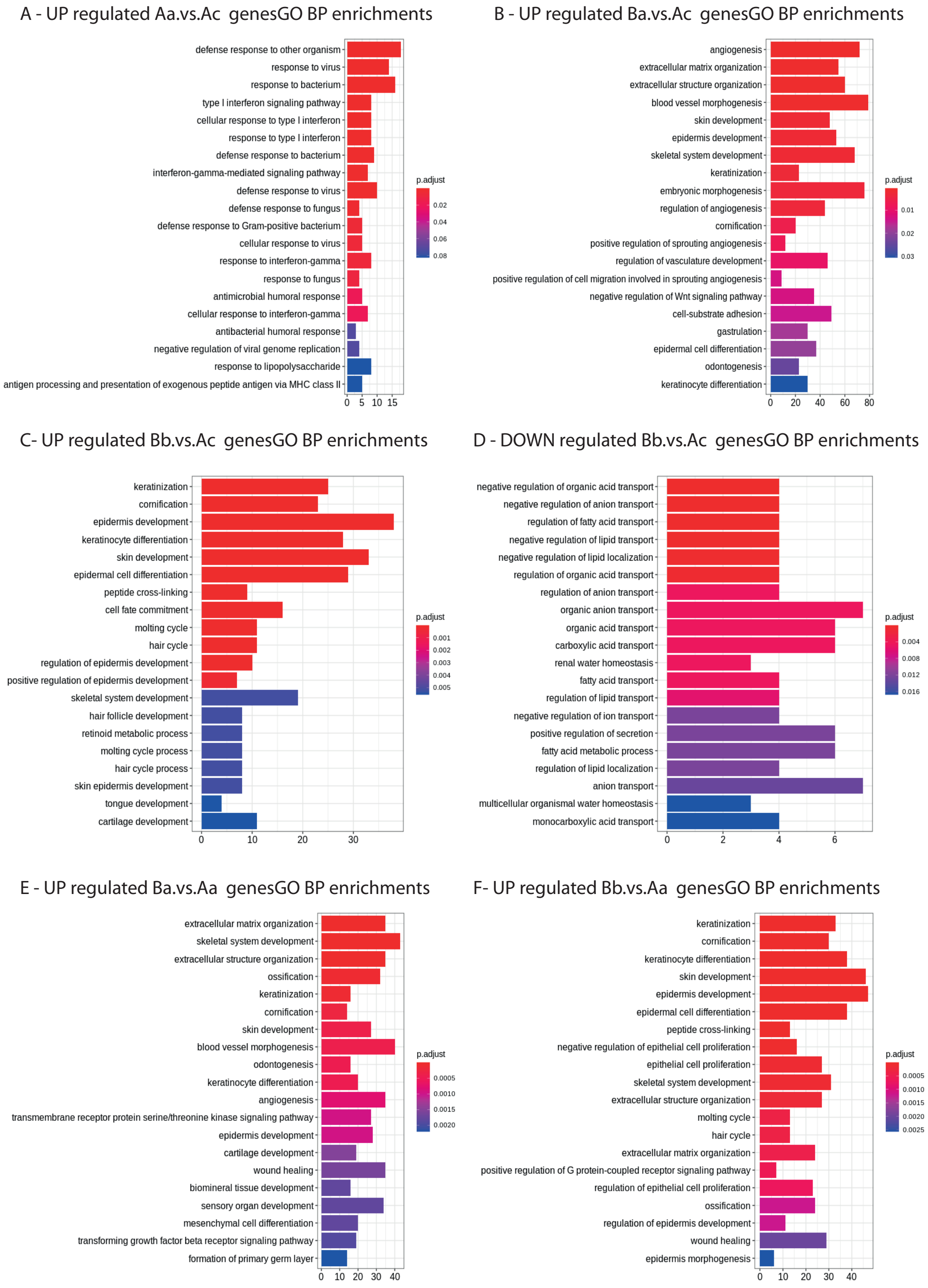

Fig. S2: **GO BP enrichments for DEG comparing CTGC**: Only comparisons that yielded statistically significant (BH adjusted p-value < 0.001) terms are shown. Specifically A CTGC-Aa vs CTGC-Ac; B CTGC-Ba vs CTGC-Ac; C and D CTGC-Bb vs CTGC-Ac; E CTGC-Ba vs CTGC-Aa and F CTGC-Bb vs CTGC-Aa. The height of the bars indicates the number of genes in the GO and color-shading the adjusted p-value.

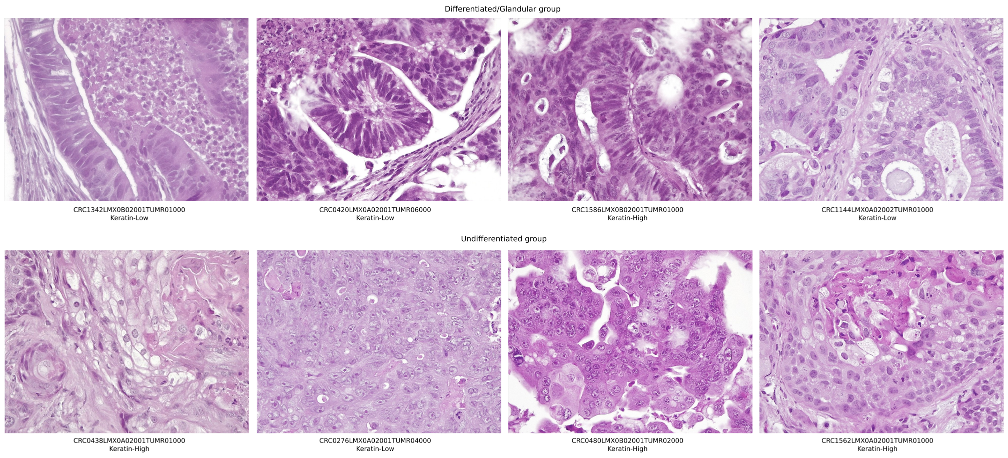

Fig. S3: **H&E morphological analysis clearly shows two distinct groups**: Haematoxilin and Eosin (H&E) was performed in blind on eight tumors from the two groups. On the top tumors are displaying histological features of a more differentiated phenotype, with clear adenomorphic structures. On the bottom, tumors are less differentiated and less structured. keratin-high and -low annotations are also shown.

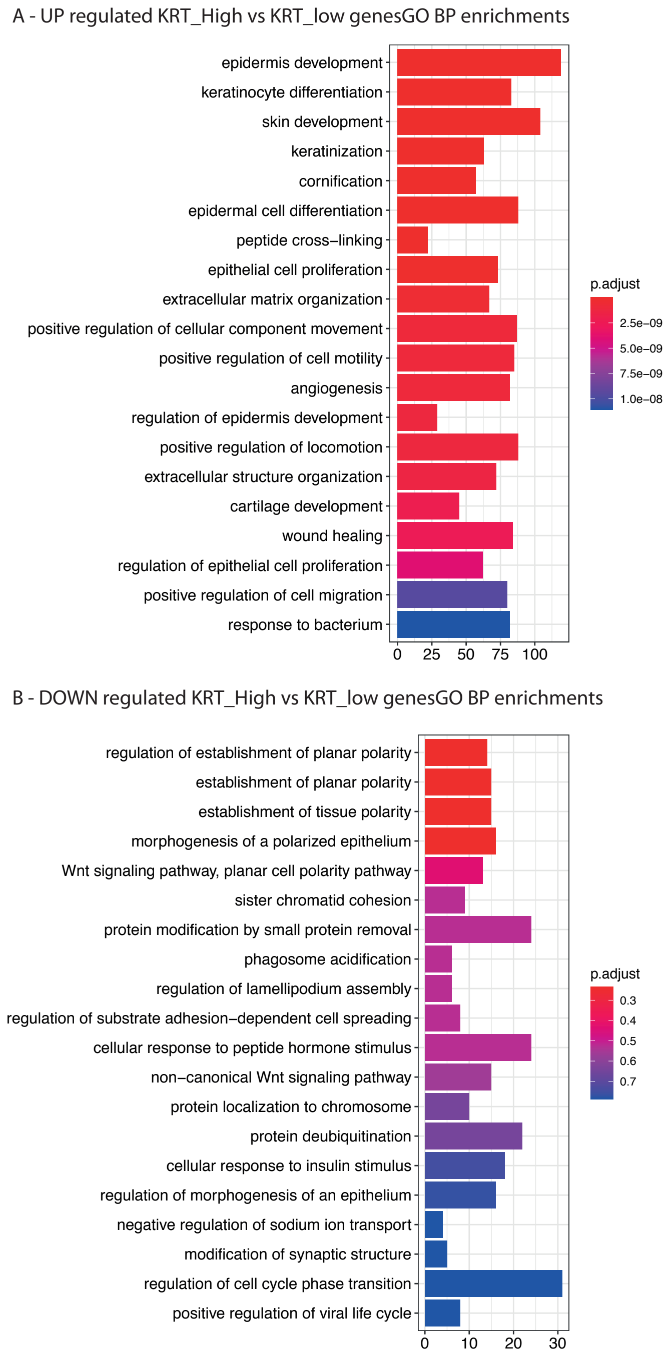

Fig. S4: **GO BP enrichments for DEG between keratin-high and keratin-low tumors**: Barplots of Gene Ontology results based on the DEG between 42 keratin-high and 15 keratin-low samples. Both upregulated (A) and downregulated (B) signatures were represented. The height of the bars indicates the number of genes in the GO and color-shading the adjusted p-value. Downregulated genes are not associated with any significant signature.

S1 Complete overview of the CONNECTOR framework

As reported in Figure 1 the CONNECTOR framework is based on the following steps:

- The *pre-processing step* consists of a visualization of the longitudinal data by a *Line plot* and a *Time grid plot* helping in the inspection of the sparsity of the time points. Specifically, in the grid of time points each point $p(x, y)$ is defined by a pair of coordinates (x, y) and by color. $p(x, y)$ is plotted only if at least one sample exists with an observation at time x and an observation at time y. Then, the grid is symmetric for the secondary diagonal. The color encodes the number of samples in which $p(x, y)$ is collected. This plot leads to visualizing which are the portions of the time grid characterized by an elevated number of measures.
- Then, by means of the FDA analysis, the sampled curves are processed by CONNECTOR with a functional clustering algorithm based on a mixed-effects model. The curves are modeled using a finite set of functions (natural cubic splines) with random effects term on the coefficients. The model is fitted and the unknown cluster membership is estimated. This step requires a *model selection phase*, in which several measures are computed to help the user properly set the two free parameters of the model. The dimension of the spline basis vector is the first free parameter to be set. Two plots, the *cross-log-likelihood* plot, and the *knots distribution* plot are generated for this task. The first is generated by the computation of the cross-log-likelihood exploiting the ten-fold cross-validation method (James *et al.*, 2000); the largest stable value of the mean cross-log-likelihood function is attained at the optimal dimension of the spline basis. The second plot visualizes the spline basis knots position for different values of the dimension. The knots divide the time domain into contiguous intervals, and the curves are fitted with separate polynomials in each interval. Hence, this plot allows the user to visualize whether the knots properly split the time domain considering the distribution of the sampled observations.

  The second free parameter is the number of clusters. The *total tightness* and the *functional Davies-Bouldin (fDB) index* are returned jointly with the stability matrices to support the parameter setting. The violin plots of the cluster dispersion (i.e. tightness) and of the cluster separation (i.e. fDB index) assist the user in the selection of the number of clusters. For the sake of completeness, for different numbers of clusters, the stability matrices are also provided to verify the consistency of the sample cluster assignments across different execution runs.
- Once the model selection phase is completed, the *output* of CONNECTOR is composed of several graphical visualizations to easily mine the results, see the bottom panel in Figure 1. The dynamics data are plotted in clusters, and for each cluster, the cluster mean curve is also reported. The curves can be colored using the features associated with each sample as reported in the Sample Data file. The discriminant plot offers a visualization of the sample separation in the CONNECTOR clusters, projected on a plane. Moreover, the discriminant function plot shows the discriminant power of each time point. Finally, the estimated curve, the confidence intervals, and the observations are reported for each sample.

The CONNECTOR software is composed of two modules consisting of (i) an R library, called CONNECTOR Package, and (ii) a docker image. Docker containerization is utilized to simplify the distribution, use and maintenance of the analysis tools. The R library provides an easier user interface for which no knowledge of the docker commands is needed.

The framework pipeline is defined by three necessary steps:

1. the data importing and processing to create the R object exploited through the entire analysis,
2. the model selection by identifying the optimal values of the two free parameters of the FCM approach (the basis spline dimension p and the number of clusters G),
3. the inspection and visualization of the obtained clusters.

In this contest, the CONNECTOR Package provides the basic functions to deal with each step of the pipeline. Finally, a step-by-step guide for installing and utilizing the framework is reported in Protocols.io available at `dx.doi.org/10.17504/protocols.io.8epv56e74g1b/v1`.

To make accessible the basic CONNECTOR functionalities even to users without expertise in R, we developed a web application through the R package Shiny RStudio, Inc (2014), a powerful R library for developing interactive web apps straight from R. Specifically, the function *RunConnectorShiny* offers a fancy web application providing a basic-level interface to configure and execute a data analysis with the CONNECTOR functionalities. Thus, users without experience in R language can directly focus on analyzing the results rather than spend their efforts setting up an R script. Therefore, the web application enables the user to run each function and go through the three steps of analysis with just a few clicks. All these functionalities make the CONNECTOR Package a flexible and easy-to-use tool.

S2 Application of CONNECTOR to PDX curves of high-grade serous epithelial ovarian cancer

To show the basic use of CONNECTOR, we analyzed the spontaneous tumor growth patterns of PDX lines derived from the propagation of one chemotherapy-naïve high-grade serous epithelial ovarian cancer (HGS-EOC), which had been passaged for five generations until the production of 21 xenografts Erriquez *et al.* (2016).

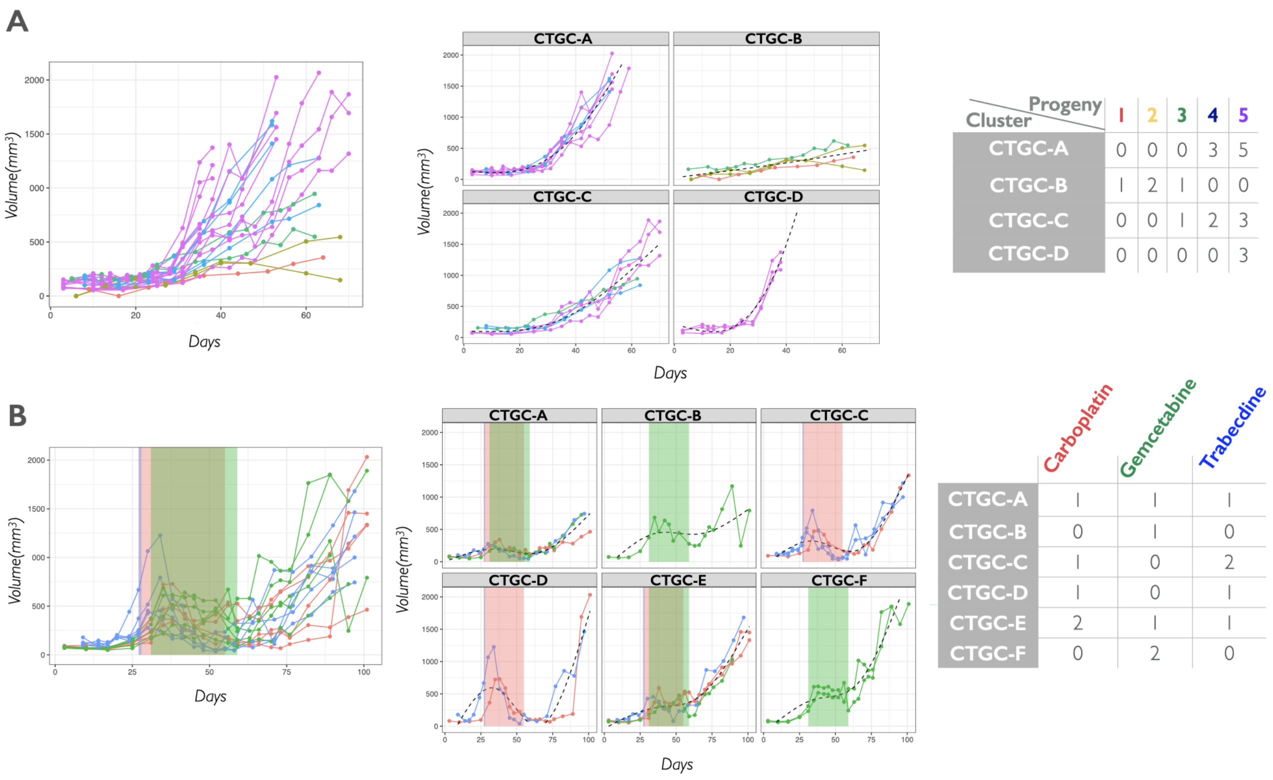

Fig. S5: **HGS-EOC PDX lines analysis. A) Untreated PDX lines.** The curves are grouped in four clusters (CTGC-A, CTGC-B, CTGC-C, CTGC-D) and the counts of the different progenies in the different clusters is reported. **B) Treated PDX lines.** The shaded boxes (red, green and blue) highlight the treatments (carboplatin, gemcitabine, and trabectedin) time windows (four weeks, four weeks, and once injection - straight blue line at day 28). The curves are grouped in six clusters (CTGC-A, CTGC-B, CTGC-C, CTGC-D, CTGC-E, CTGC-F); the counts of the PDX lines for each treatment in the CTGCs are reported.

The progression curves of the PDX lines are reported in Figure S5A. The time grid suggests that until 70 days the frequency of the observations is larger than 0.75 and it drops for larger times, so we truncated the curves at day 70. The model selection suggests that the optimal parameters set are dimensions of the spline basis equal to 3 and the number of clusters equal to 4 (CTGC-A, CTGC-B, CTGC-C, CTGC-D). This combination of the parameters led to an average value of cluster stability equal to 0.8. The detailed CONNECTOR analysis is reported in Figure S6. Figure S5A shows the four CTGCs in which it is possible to appreciate four different progression patterns.

Notably, the cluster aggregation reflected a generation separation, likely due to intra-tumor heterogeneity. Indeed, initial engraftment is expected to impose a selection bottleneck, and subsequent propagations exacerbate clonal divergence owing to repeated sampling bias and genomic evolution. During the growth of each PDX line, functional outliers came out, that is, single PDXs emerged that were characterized by increased growth rates. For example, the PDX lines in CTGC-D reached $1000mm^3$ of volume in 40 days with respect to the models in CTGC-A or CTGC-C, which reached the same volume in 60 and 70 days, respectively. These outliers might represent the expansion of clonal subpopulations with a fitness advantage. Moreover, the discriminant function returned by CONNECTOR highlights the strong discriminant power of the first 15 days of observation, which can be ascribed to the intra-tumor heterogeneity, see Figure S6. The comparisons of the CONNECTOR performance with respect to the classical fitting methods followed by k-means clustering and other functional methods are reported below.

To establish the versatility of CONNECTOR in analyzing different dynamics, we investigated the tumor growth curves of 15 PDX lines propagated from the same chemotherapy-naive HGS-EOC sample presented above, which were exposed to carboplatin (n=5), gemcitabine (n=5) or trabectedin (n=5). Carboplatin and gemcitabine were administered twice weekly for 4 weeks via intraperitoneal injection; trabectedin was only once administered through the tail vein. The optimal model selected indicates the dimension of the spline basis equals 4 and the number of clusters equals to 6 (CTGC-A, CTGC-B, CTGC-C, CTGC-D, CTGC-E, CTGC-F); considering this setting the cluster stability is 0.9. The detailed CONNECTOR analysis is reported in Figure S7.

In this cohort of PDXs, the response to drugs of individual models was uneven; some models showed a significant reduction, while other models proved to be resistant, Figure S5B. Notably, the discriminant function reveals that the time with the highest discriminatory power corresponds to day 35.

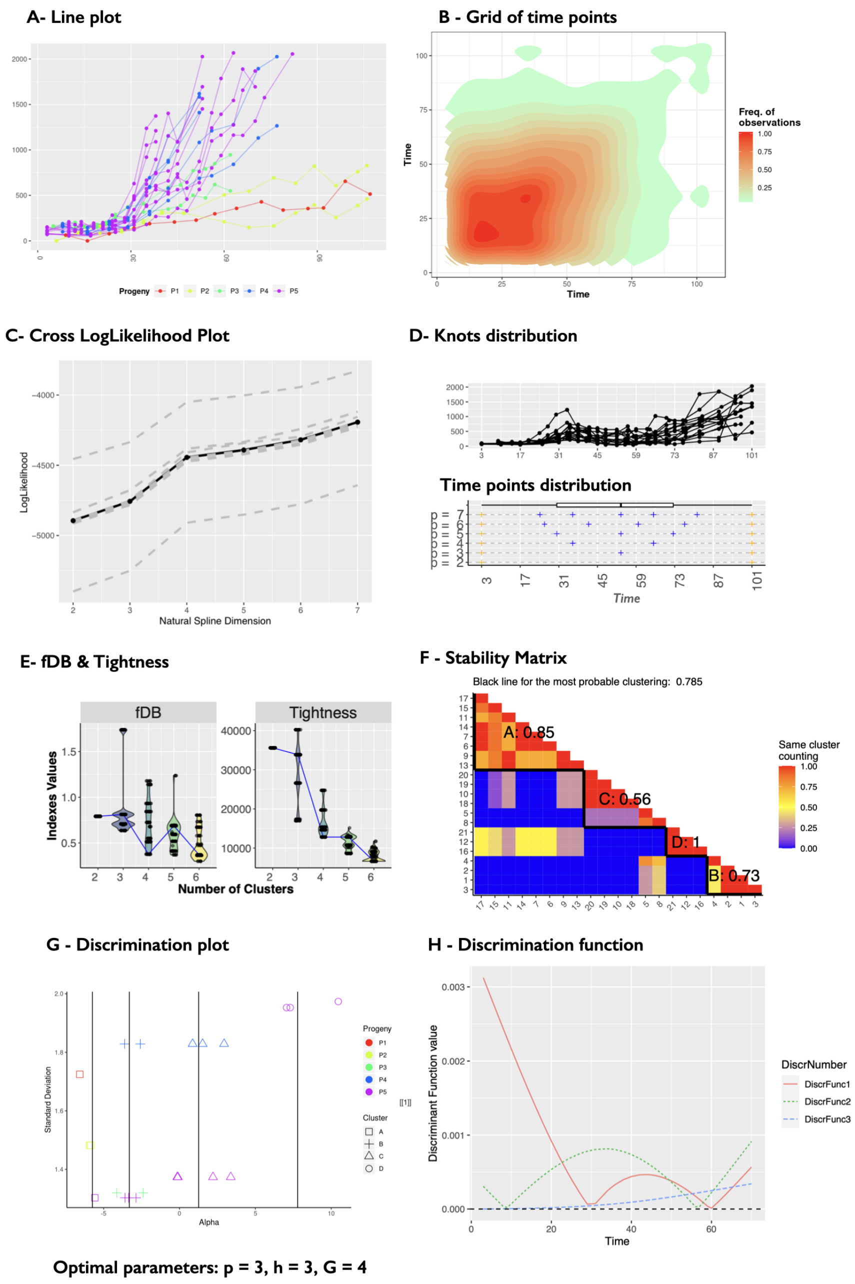

Fig. S6: **A** line plot of the 21 PDX curves of HGS-EOC tumor. **B** Time grid, the suggested time at which truncate the data is 70 days. **C** Cross LogLikelihood plot reports 4 as the optimal parameter for the base spline, this value is also supported by the Knots distribution, **D**. **E** fDB and tightness violin plot for the selection of the number of clusters, fours are associated with the lower fDB value, and at 4 is appreciable the elbow in the tightness plot. **F** the stability matrix obtained selecting 4 clusters. **G** Discriminant plot and **H** discriminant functions. The optimal set of parameters selected is p=3, h=3, and G=4.

Indeed, around that time, the curves start to be separated as a consequence of the chemotherapy treatments effects. In detail, in CTGC-A, CTGC-B, and CTGC-F, the tumors remained stable during the treatment time window, while in CTGC-C and CTGC-D a clear increment, followed by a reduction of the tumor mass, was appreciable. This variable distribution of responses can be explained, again, with a high degree of intra-tumor heterogeneity in the original tumor from which the different PDX lines were propagated; indeed, the genetic deviation is expected to influence response to therapy Schmitt *et al.* (2016).

We illustrated the versatility of CONNECTOR starting with the analysis of the spontaneous growth patterns in 21 PDX lines propagated from a single HGS-EOC tumor sample. We observed uneven growth rates in PDX lines derived from the same original tumor. This is consistent with the notion that ovarian cancers show a high degree of intratumor heterogeneity McPherson *et al.* (2016)Schwarz *et al.* (2015)Castellarin *et al.* (2013), which results in

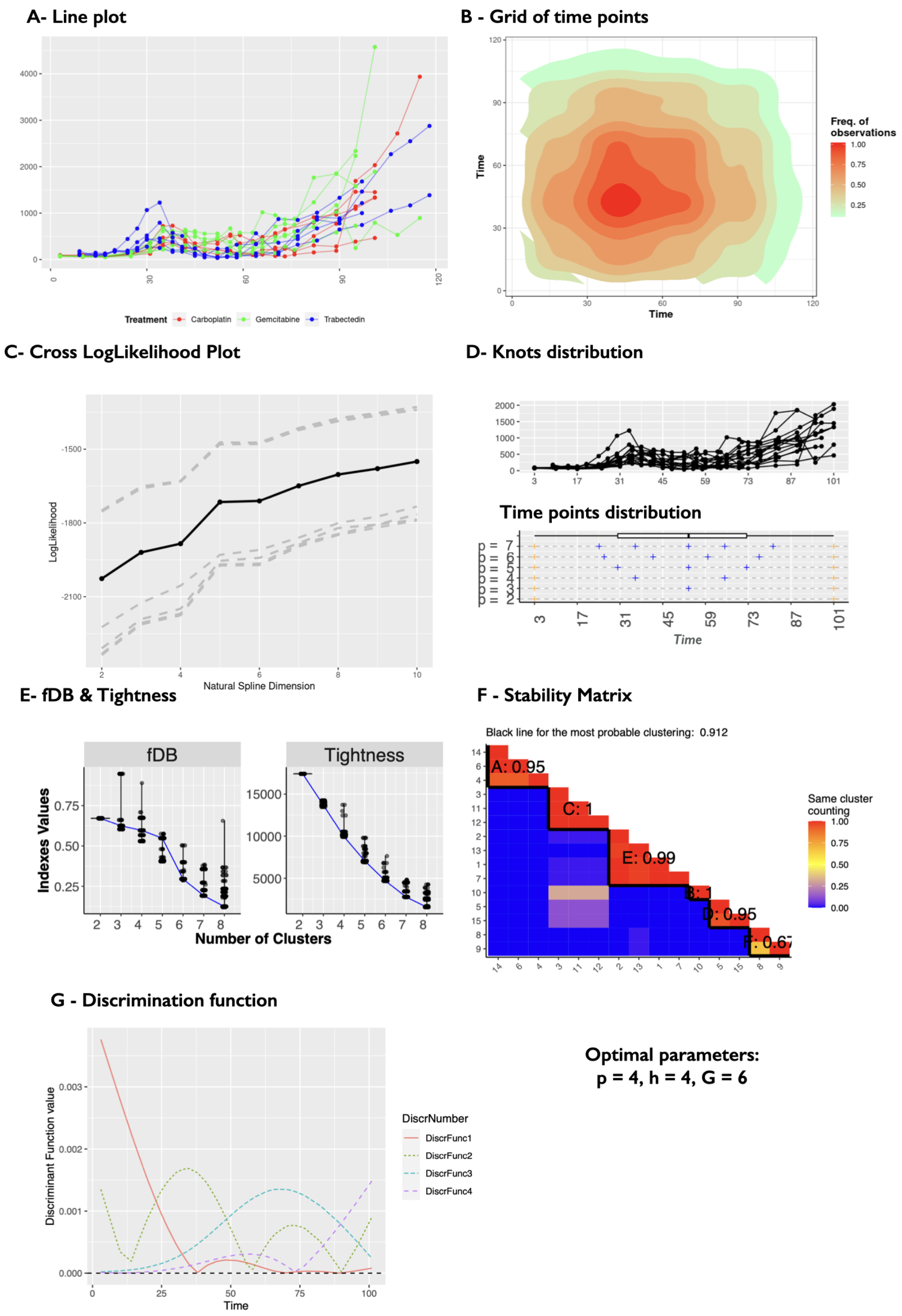

Figure 31: Discriminant curve.

Fig. S7: **A** line plot of the 25 PDX curves of HGS-EOC tumor treated with three drugs treatments, carboplatin, gemcitabine and trabectedin, reported in red, green, and blue, respectively. **B** Time grid, the suggested time at which truncate the data is 70 days. **C** Cross LogLikelihood plot reports 4 as the optimal parameter for the base spline, this value is also supported by the Knots distribution, **D**. **E** fDB and tightness violin plot for the selection of the number of clusters, six is associated with the lower fDB value. At 6 is appreciable a slight elbow in the tightness plot. **F** the stability matrix obtained by selecting 6 clusters. **G** the discriminant functions are reported. The optimal set of parameters selected is p=4, h=4, and G=6.

the establishment of genetically different tumor entities during serial propagation. This diversification can also explain the varied responses to treatment observed in 15 PDX lines – again derived from the same original tumor – exposed to different chemotherapeutics.

S3 Application of CONNECTOR to PDX curves of metastatic colorectal cancer: details

Inspection of CONNECTOR results The CONNECTOR results on all PDX growth curves of metastatic colorectal cancer reveal that 3 clusters is the optimal solution. We then investigated the sub-clustering of the CONNECTOR clusters A and B. The fDB and tightness plots of Cluster A highlighted that 3 and 5 clusters are both reasonable choices. Otherwise in cluster B, 3 resulted as the optimal number of clusters. Moved by the deep nature of clustering solutions that cannot be evaluated in a problem-independent way, we reported a comparison between two alternative scenarios: *scenario (i)* where all curves are divided into 3 clusters, namely A, B, and C; Cluster A is divided into 5 groups (Aa, Ab, Ac, Ad, and Ae), and Cluster B is divided into 3 groups (Ba, Bb, and Bc) reaching in total 9 CTGCs. *scenario (ii)* all curves are divided into 3 clusters, namely A, B, and C; Cluster A is divided into 3 sets (Aa, Ab, and Ac), and Cluster B is divided into 3 sets (Ba, Bb, and Bc) reaching in total 7 CTGCs.

The results of *scenario (i)* are reported in sections 3.1 and 3.2 of the main paper.

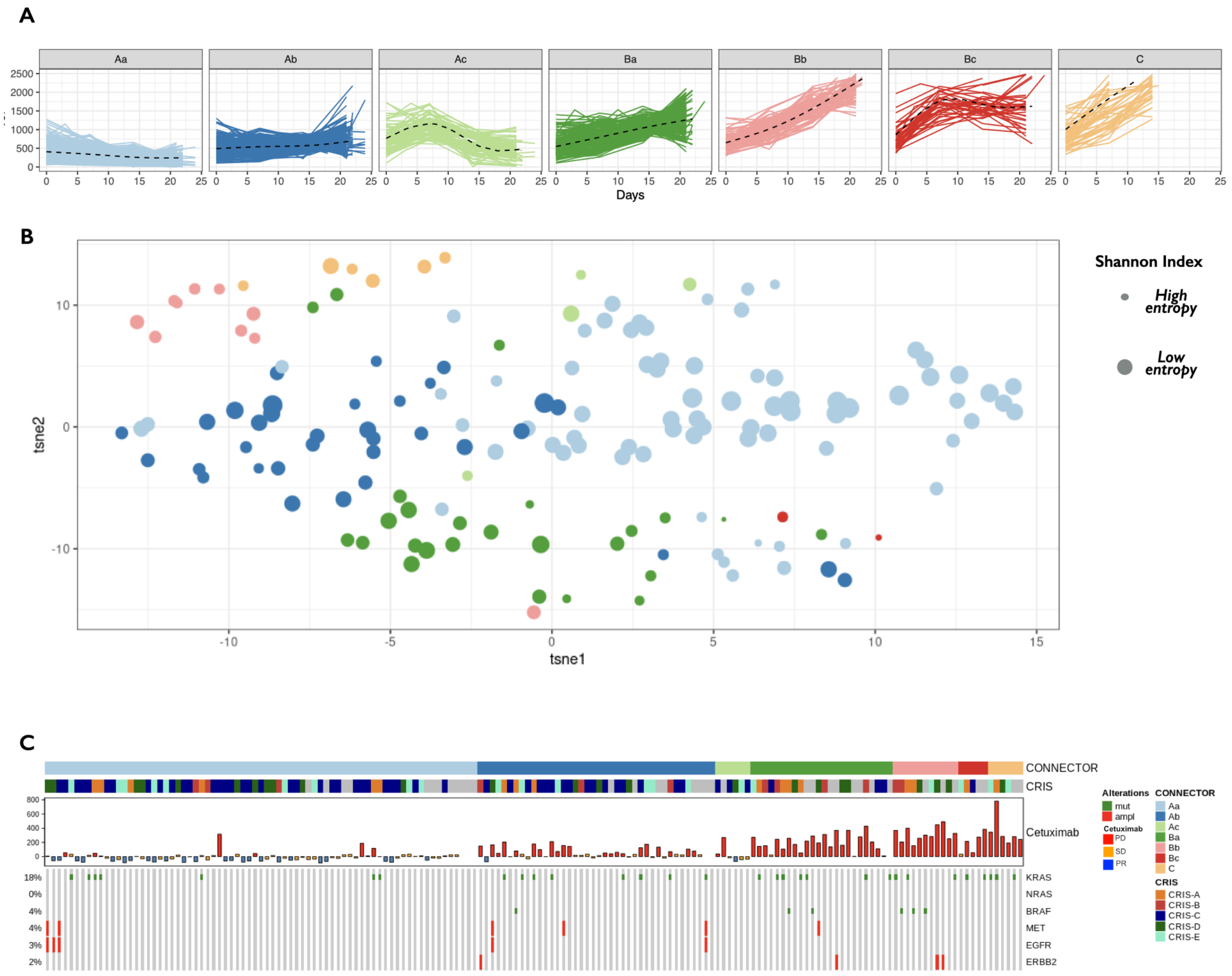

Fig. S8: **A) CONNECTOR tumor growth classes.** The seven boxes result from a first run with a number of clusters equal to 3 followed by second runs on the CTGC-A (with 3 sub-classes) and on the CTGC-B (with 3 sub-classes). **B) t-SNE visualization of the CTGCs induced on the parental tumors.** Each dot corresponds to a parental tumor. The color of the dots matches the color of the assigned CTGC, see Panel A. The dimension of the dots is inversely proportional to the Shannon Index calculated on the distribution of the curves of the same parental tumor across CTGCs (large dots - small entropy). **C) Molecular and phenotypic characterization of the CONNECTOR clustered mCRC xenografts.** Each sample was annotated according to CRIS subtype, response to cetuximab, and somatic alteration known to determine cetuximab resistance or sensitivity.

Concerning *scenario (ii)* seven plots of the tumor growth clusters are reported in Figure S8 Panel A. In the scatter plot, reported in Figure S8 Panel B, it is appreciable that distinctly isolated CTGCs, by focusing on the color and the size of the dots, suggest their coherence. In this scenario, the new CTGCs are also characterized by strong biological correlates, see Figure S8 Panel C, with a polarization of non-responder tumors in clusters Ab, Ba, Bb, Bc, and C and a strong enrichment of partial responses (PR) and growth controls (SD) in Aa. CRIS-C is associated with Aa and well-known resistance markers are over-represented in CTGCs Ba and Bb. Moreover, differential gene expression analyses indicate a strong keratinization signal in Ba and Bb, as expected.

Comparing with *scenario (i)* to *scenario (ii)*, it is appreciable that the division of cluster A in five CTGCs (*scenario (i)*) presents a higher granularity resulting in A subclusters being more specific, with one enriched in PR, one in SD, and one in PD, while the division of cluster A in three CTGCs (*scenario (ii)*) tends to split the SD between Aa and Ab and thus dilute the signal.

mCRX PDX generation and treatment protocol After surgical removal from patients, each metastatic colorectal cancer specimen was fragmented and either frozen or prepared for implantation: cut into small pieces of which 2 fragments were implanted in 2 mice. After engraftment and tumor mass formation, the tumors were passaged and expanded for 2 generations until the production of 2 cohorts, each consisting of 12 mice. Tumor size was evaluated once weekly by calliper measurements and the approximate volume of the mass was calculated using the formula $4/3\pi(d/2)^2 D/2$, where d is the minor tumor axis and D is the major tumor axis. PDXs derived from each original fragment were then randomized for treatment with placebo (6 mice) or cetuximab (6 mice); animals with established tumors, defined as an average volume of $400mm^3$, were then treated with cetuximab (Merck, White House Station, NJ) 20 mg/kg/twice-weekly i.p.

For assessing CTGCs response to therapy, we used averaged volume measurements at 3 weeks after treatment normalized to the tumorgraft volume at the time of cetuximab treatment initiation. Tumors are then classified as follows:

1. *partial response* (**PR**): decrease of at least 50% in tumor volume
2. *progressive disease* (**PD**): increase of at least a 35% in tumor volume
3. *stable disease* (**SD**): the ones between 50% decrease and 35% increase

All animal procedures were approved by the Ethical Commission of the Institute for Cancer Research and Treatment and by the Italian Ministry of Health.

Transcriptional analyses Differentially expressed genes (DEGs) between different CTGC or keratin-high and -low groups were obtained using the R package DESeq2 (v1.26.0) Love *et al.* (2014) with design: batch+cluster. Here batch is used to correct for sequencing batches and cluster to select samples belonging to the CTGC of interest (or keratin-high and -low). We did not compare CTGC clusters for which less than 5 samples were available. Genes with more than 5 reads in only 1 sample were removed before testing for differential expression, DEGs were identified using |LFC| >=0.5849625 and adjusted p-value < 0.05. Gene Ontology analyses for the upregulated or downregulated genes were performed with the R library ClusterProfiler (v3.14.3) Wu *et al.* (2021); Yu *et al.* (2012).

The selection of a robust set of markers for the epithelial stratification differentiation phenotype was performed with two criteria:

- being assigned to the GO keratinization (GO:0031424 Sup (2022)), the most significantly enriched term across all the CTGC-Ac vs other CTGC comparisons, via direct experimental evidence, using filters for the taxon (Homo Sapiens) and evidence (manual assertion) on the EBI Quick GO web site.
  AND

- being significantly upregulated either in Bb versus Ac or Bc versus Ac

The resulting genes are: KRT80, KRT7, KRT86, KRT81, KRT83, KRT6B, KRT6A, KRT74, KRT79, KRT16. To classify samples in high or low expressing keratin groups, quartiles of the expression level of the ten selected genes were calculated from progressive disease (PD) samples belonging to CTGC-Ac. Each sample was then assigned ten labels, according to its expression of those keratins with respect to CTGC-Ac first and third quartiles - “high” if the expression is larger than the 3rd quartile and “low” if it is smaller than the 1st. Samples were then classified as “keratin-high” if they were assigned no low labels, and “keratin-low” if they were assigned no high labels.

Z transformed expression values in COAD/READ patients for HOPX were downloaded alongside clinical annotations from CBioPortal (Firehose legacy dataset), and patients with multiple expression values or missing expression/survival data were filtered out. Survival analysis was performed on the remaining 371 patients using the libraries survival and survminer Therneau (2022); Kassambara (2021), and the optimal threshold for HOPX expression was determined using the maximally selected rank statistic approach (maxstat, Hothorn (2017)), correcting accordingly the resulting log rank p value. DEG and all the enrichment and survival analyses were performed with R version 3.6.3.

Morphological analyses Haematoxilin and Eosin was performed in xenografts from mice treated with vehicle (until tumors reached an average volume of $1500mm^3$). Tumors were explanted, routinely processed and stained with H&E (Bio-optica). Images were captured with the Leica LAS EZ software using a Leica DM LB microscope.

S4 Further computational methods

Naive Bayesian Classification The *naive Bayesian classification* is a probabilistic classifier based on the Bayes’ theorem. It can be efficiently exploited to assign a cluster membership to each parental tumor (from now on referred to model) given the cluster membership of every curve of the PDXs derived from it. Let $(k_1^i, \dots, k_{n_i}^i)$ be the clusters assigned to the n_i PDX curves belonging to the ith model. Thus, the classifier assigns the cluster $\hat{k_i} \in \{1, \dots, G\}$ to the ith model as:

$$\hat{k_i} = \underset{k \in \{1,\dots,G\}}{\text{argmax}} \frac{n_i^k}{n_i}, \tag{S1}$$

where $\frac{n_i^k}{n_i}$ is the frequency of having n_i^k curves of the ith model in the kth cluster.

Shannon Index The *Shannon index* Shannon (1948) is an information statistic index that we used to quantify curve diversity in a specific PDX model. It is computed as follows:

$$H_i = -\sum_{k=1}^{G} p_{k,i} \log_2 p_{k,i}, \tag{S2}$$

where H_i is the Shannon index for the ith model, G is the number of clusters, $p_{k,i}$ is the proportion of PDX curves from the ith model and belonging to the kth cluster. Therefore, a larger value of H_i corresponds to a larger diversity of the ith PDX model, i.e. many different clusters are assigned to the same model. If H_i is equal to zero it means that the curves of the ith PDX model belong to the same cluster.

t-distributed stochastic neighbor embedding (t-SNE) t-SNE Van der Maaten and Hinton (2008) is a statistical method used to visualize high-dimensional data in a two or three-dimensional map exploiting a nonlinear dimensionality reduction technique. t-SNE is performed on a dataset containing for each model the frequency distribution of the PDX curves over the CTGCs considered, that is a vector sized the number of CTGCs number reporting the proportion of PDX curves belonging to each CTGC. The scatter plot generated provided a spatial visualization of the agreement of the PDX curves belonging to a given model. Indeed, the size of the dot corresponds to the grade of agreement (in terms of the Shannon Index) of all PDX curves belonging to that model while the color corresponds to a specific CTGC.

S5 Model Selection Tools: numerical details.

The parametrized family of semi-metrics between curves, denoted as D_q and defined in eq. (3), is the building block for both the *total tightness* T defined in eq. (4) and the *functional* DB (fDB) defined in eq. (5). Such indexes are proposed as measures for the quality of the clustering of functional data, useful for a proper comparison between different classifications, and as a guide to the user. Let's here illustrate the numerical details needed for their reliable calculation.

Following Ferraty and Vieu (2006b), the numerical evaluation of D_q should exploit the spline approximation of the curves involved. For $q = 0$ the curves are not differentiated and hence equations. (S4) and (S3) give the values of the curves at any points. Hence, the integral in eq. (3) can be evaluated using an appropriate quadrature formula on the full-time grid, made of all the sampled time points for all the sampled curves, as CONNECTOR calculates curve predictions and cluster means for the entire time interval.

For $q \geq 1$, the computation of successive derivatives is performed by direct calculation of their analytical form. More precisely, the estimated i–th curve is given by eq. (S4) as a linear combination of the spline basis functions $\mathbf{s}(t)$ (with known analytical expressions) and the coefficients $\hat{\boldsymbol{\eta}}$

$$\hat{g}_i(t) = \mathbf{s}(t)^T \hat{\boldsymbol{\eta}}_i,$$

and the differentiated estimated i–th curve can be represented as

$$\hat{g}_i^{(1)}(t) = \frac{d}{dt}\hat{g}_i(t) = \frac{d}{dt}\mathbf{s}(t)^T \hat{\boldsymbol{\eta}}_i = \mathbf{s}^{(1)}(t)^T \hat{\boldsymbol{\eta}}_i,$$

where the analytical expressions of $\mathbf{s}^{(1)}(t)$ are known too, being the derivatives of the basis functions $\mathbf{s}(t)$. Let us comment that, the functional clustering method James and Sugar (2003) considers the spline basis $\mathbf{s}(t)$ obtained by the singular value decomposition of the B-spline basis for a natural cubic spline. Hence the (k, p) matrix A with elements the values of the B-spline basis (dimension p) functions evaluated at the grid time points (k) is decomposed as

$$A = U \cdot D \cdot V^t,$$

where U and V are (k, k) and (p, p) orthogonal matrices and D is a (k, p) diagonal with non zero elements $(p_1, \ldots, p_p)$. The spline basis considered in James and Sugar (2003) is given by the first p columns of the matrix U (the only columns which enter the product with matrix D). Hence for $i = 1, \ldots, p$, the i-th column of such matrix is given by the linear transformation

$$U_i = \frac{1}{p_i}(A \cdot V)_i.$$

When $q \geq 1$, the same linear transformation applies to the matrix B with elements the q-th derivative of the natural B-spline basis functions evaluated at the grid time points. So finally the integral in definition (3) can be evaluated with a suitable quadrature formula.

S6 Functional Clustering Model: details and fitting algorithm

Notice that $\boldsymbol{\lambda}_0$, Λ and $\boldsymbol{\alpha}_k$ are confounded if no constraints are imposed. Hence, we ask

$$\sum_k \boldsymbol{\alpha}_k = 0,$$

meaning that $\mathbf{s}(t)^T \boldsymbol{\lambda}_0$ may be interpreted as the overall mean curve. Moreover, we ask

$$\Lambda^T S^T (\sigma^2 I + S \Gamma S^T)^{-1} S \Lambda = I,$$

the reason of which is well explained in James and Sugar (2003).

Notice that the kth cluster mean curve can be retrieved as

$$\bar{g}^k(t) = \mathbf{s}(t)^T(\hat{\boldsymbol{\lambda}}_0 + \hat{\Lambda}\hat{\boldsymbol{\alpha}}_k). \tag{S3}$$

Moreover, the functional clustering procedure can accurately predict unobserved portions of the curves $g_i(t)$ by means of the natural estimate

$$\hat{g}_i(t) = \mathbf{s}(t)^T\hat{\boldsymbol{\eta}}_i, \tag{S4}$$

where $\hat{\boldsymbol{\eta}}_i$ is a prediction for $\boldsymbol{\eta}_i$ which is proven to be optimally computed as $\mathbb{E}(\boldsymbol{\eta}_i \mid \mathbf{Y}_i)$ and explicitly given in James and Sugar (2003), eq. (17).

S6.1 The fitting algorithm

Fitting the model consists of estimating the parameters $\boldsymbol{\lambda}_0, \Lambda, \boldsymbol{\alpha}_k, \Gamma$ and σ^2, included in eq. (2). The cluster membership is treated as missing data through a multinomial random variable $\boldsymbol{z}_i$ with parameters $\boldsymbol{\pi} = (\pi_1, \ldots, \pi_G)$, where π_k is the probability that the observation belongs to cluster k.

As the cluster memberships $\boldsymbol{z}_i$'s and the $\boldsymbol{\gamma}_i$'s are assumed to be independent, the complete log-likelihood can be factorized as follows:

$$l(\boldsymbol{\lambda}_0, \Lambda, \boldsymbol{\alpha}_k, \Gamma, \sigma^2, \boldsymbol{\pi}) = \sum_{i=1}^{n}\sum_{k=1}^{G} z_{ik} \cdot \log(\pi_k) + \tag{S5}$$

$$-\frac{n}{2}\log(\det(\Gamma)) - \frac{1}{2}\sum_{i=1}^{n}\boldsymbol{\gamma}_i^T\Gamma^{-1}\boldsymbol{\gamma}_i + \tag{S6}$$

$$-\frac{1}{2}\sum_{i=1}^{n}\sum_{k=1}^{G} z_{ik}\Big[n_i\log(\sigma^2) + \frac{1}{\sigma^2}(\boldsymbol{Y}_i - S_i(\boldsymbol{\lambda}_0 + \Lambda\boldsymbol{\alpha}_k + \boldsymbol{\gamma}_i))^T(\boldsymbol{Y}_i - S_i(\boldsymbol{\lambda}_0 + \Lambda\boldsymbol{\alpha}_k + \boldsymbol{\gamma}_i))\Big]. \tag{S7}$$

The equation displays the $\boldsymbol{z}_i$'s log-likelihood in the first row (S5), the $\boldsymbol{\gamma}_i$'s log-likelihood in the second row (S6), which are $\sim N(0, \Gamma)$, and the $\boldsymbol{Y}_i$'s conditional log-likelihood in the last two rows (S7), which are $\sim N(S_i \cdot (\boldsymbol{\lambda}_0 + \Lambda\boldsymbol{\alpha}_k), \sigma^2 I)$.

The EM algorithm is ruled to iteratively maximize the expected values of (S5), (S6) and (S7), given $\boldsymbol{Y}_i$ and the current parameter estimates. Hence the algorithm iterates through the following steps:

1. **Compute the membership probabilities.** As the z_{ik} are actually unknown, we calculate their expected values by Bayes theorem

$$\pi_{k|i} = \mathrm{P}(z_{ik} = 1 \mid \boldsymbol{Y}_i) = \frac{f_k(y)\pi_k}{\sum_{j=1}^{G} f_j(y)\pi_j}, \tag{S8}$$

   where f_k is the conditional density of the $i-$th curve belonging to the $k-$th cluster, that is multivariate gaussian with mean vector $S_i(\boldsymbol{\lambda}_0 + \Lambda\boldsymbol{\alpha}_k)$ and covariance matrix $\sigma^2 I + S_i\Gamma S_i^T$, see eq. (2).
2. **E-M steps for** (S5)**.** In the E-step the expected log-likelihood is computed by substituting z_{ik} for $\pi_{k|i}$ and in the M-step this quantity is maximized. The result can be explicitly calculated:

$$\pi_k = \frac{1}{n}\sum_{i=1}^{n}\pi_{k|i} \tag{S9}$$

3. **E-M steps for** (S6)**.** The $\boldsymbol{\gamma}_i$'s are multivariate Gaussian with zero mean vector and covariance matrix Γ and the $\boldsymbol{Y}_i$'s, conditional on the i-th curve belonging to the k-th cluster, are multivariate Gaussian as well, with mean vector $\mu_{Y_{ik}}$ and covariance matrix $\Sigma_{Y_{ik}}$ given as follows

$$\mu_{Y_{ik}} = S_i \cdot (\boldsymbol{\lambda}_0 + \Lambda\boldsymbol{\alpha}_k)$$
$$\Sigma_{Y_{ik}} = \sigma^2 I + S_i\Gamma S_i^T.$$

   Hence the conditional distribution of the $\boldsymbol{\gamma}_i|\boldsymbol{Y}_i, z_{ik} = 1$ is again Gaussian with mean vector $\mu_{\gamma_{ik}}$ and covariance matrix $\Sigma_{\gamma_{ik}}$ given as follows

$$\mu_{\gamma_{ik}} = (\sigma^2\Gamma^{-1} + S_i^T S_i)^{-1}S_i^T \cdot (\boldsymbol{Y}_i - S_i\boldsymbol{\lambda}_0 - S_i\Lambda\boldsymbol{\alpha}_k)$$
$$\Sigma_{\gamma_{ik}} = (\Gamma^{-1} + S_i^T S_i/\sigma^2)^{-1}.$$

   The above equations are obtained considering that the covariance matrix of the $\boldsymbol{\gamma}_i$'s and $\boldsymbol{Y}_i$'s is $(S_i\Gamma)^T$. Hence the expectation of (S6) is maximized by the sample covariance matrix

$$\begin{aligned}\hat{\Gamma} &= \frac{1}{n}\sum_{i=1}^{n}\mathbb{E}(\boldsymbol{\gamma}_i\boldsymbol{\gamma}_i^T|\boldsymbol{Y}_i) \\ &= \frac{1}{n}\sum_{i=1}^{n}\sum_{k=1}^{G}\pi_{k|i}\mathbb{E}(\boldsymbol{\gamma}_i\boldsymbol{\gamma}_i^T|\boldsymbol{Y}_i, z_{ik} = 1) \\ &= \frac{1}{n}\sum_{i=1}^{n}\sum_{k=1}^{G}\pi_{k|i}\Sigma_{\gamma_{ik}}\end{aligned} \tag{S10}$$

4. **E-M steps for** (S7)**.** The last part of the complete log-likelihood contains the parameters $\boldsymbol{\lambda}_0$, $\boldsymbol{\alpha}_k$, Λ and σ^2. The expectation is repeatedly maximized until convergence, calculating explicitly the following point of maxima, for $\boldsymbol{\lambda}_0$ and $\boldsymbol{\alpha}_k$:

$$\hat{\boldsymbol{\lambda}}_0 = \left(\sum_{i=1}^{n} S_i^T S_i\right)^{-1} \sum_{i=1}^{n} S_i^T \left(\boldsymbol{Y}_i - \sum_{k=1}^{G} \pi_{k|i} S_i (\Lambda \boldsymbol{\alpha}_k + \mu_{\gamma_{ik}})\right), \tag{S11}$$

$$\hat{\boldsymbol{\alpha}}_k = \left(\sum_{i=1}^{n} \pi_{k|i} \Lambda^T S_i^T S_i \Lambda\right)^{-1} \sum_{i=1}^{n} \pi_{k|i} \Lambda^T S_i^T \left(\boldsymbol{Y}_i - S_i(\hat{\boldsymbol{\lambda}}_0 + \mu_{\gamma_{ik}})\right), \tag{S12}$$

and for each column of Λ:

$$\hat{\Lambda}_c = \left(\sum_{i=1}^{n}\sum_{k=1}^{G} \pi_{k|i} \hat{\alpha}_{k,c}^2 S_i^T S_i\right)^{-1} \sum_{i=1}^{n}\sum_{k=1}^{G} \pi_{k|i} \hat{\alpha}_{k,c} S_i^T \left(\boldsymbol{Y}_i - S_i \hat{\boldsymbol{\lambda}}_0 - \sum_{r \neq c} \hat{\alpha}_{k,r} S_i \hat{\Lambda}_r - S_i \mu_{\gamma_{ik}}\right), \tag{S13}$$

where $\hat{\alpha}_{k,c}$ is the c-th component of $\hat{\boldsymbol{\alpha}}_k$. Finally the expected log-likelihood is maximized for σ^2, at the point:

$$\begin{aligned}\hat{\sigma}^2 &= \frac{1}{\sum_{i=1}^{n} n_i} \sum_{i=1}^{n}\sum_{k=1}^{G} \pi_{k|i} \mathbb{E}\left[(\boldsymbol{Y}_i - S_i(\hat{\boldsymbol{\lambda}}_0 + \hat{\Lambda}\hat{\boldsymbol{\alpha}}_k + \boldsymbol{\gamma}_i))^T (\boldsymbol{Y}_i - S_i(\hat{\boldsymbol{\lambda}}_0 + \hat{\Lambda}\hat{\boldsymbol{\alpha}}_k + \boldsymbol{\gamma}_i)) | \boldsymbol{Y}_i, z_{ik} = 1\right] \\ &= \frac{1}{\sum_{i=1}^{n} n_i} \sum_{i=1}^{n}\sum_{k=1}^{G} \pi_{k|i} \left[(\boldsymbol{Y}_i - S_i(\hat{\boldsymbol{\lambda}}_0 + \hat{\Lambda}\hat{\boldsymbol{\alpha}}_k + \mu_{\gamma_{ik}}))^T (\boldsymbol{Y}_i - S_i(\hat{\boldsymbol{\lambda}}_0 + \hat{\Lambda}\hat{\boldsymbol{\alpha}}_k + \mu_{\gamma_{ik}})) + S_i \Sigma_{\gamma_{ik}} S_i^T\right] \end{aligned} \tag{S14}$$

The fitting algorithm is outlined in the box 1. Notice that the initial guess for the cluster memberships of the curves is calculated through a k-means clustering algorithm on the spline coefficients $\boldsymbol{\eta}_i$, see eq. (1), calculated as $(S_i^T S_i)^{-1} S_i^T \boldsymbol{Y}_i$.

Algorithm 1: The fitting algorithm

Data: $\boldsymbol{Y}_i, p, h, G, S_i$
Result: $\pi_k, \Gamma, \lambda_0, \alpha_k, \Lambda, \sigma^2$; `/* log likelihood maximization */`

1. Take initial guesses for $\pi_k, \Gamma, \lambda_0, \alpha_k, \Lambda, \sigma^2$;
2. *Expectation Step*: compute the membership probabilities π_k using equations (S9) and (S8);
3. *Maximization Step*:
   - 3.a compute $\hat{\Gamma}$ given in eq. (S10);
   - 3.b iteratively until convergence compute $\hat{\boldsymbol{\lambda}}_0$, $\hat{\boldsymbol{\alpha}}_k$ and $\hat{\Lambda}_c$ given in equations (S11), (S12) and (S13);
   - 3.c compute $\hat{\sigma}^2$ given in eq. (S14);
4. Iterate steps (2) and (3) until all the parameters have converged.

S7 CONNECTOR: evaluation of the performances

S7.1 Accuracy of the model selection procedure

In this Section, we illustrate a simulation study with the purpose to point out the performances of CONNECTOR for different choices of the free parameters p, the spline basis dimension, and G, the number of clusters.

We run CONNECTOR on a simulated dataset, made of points sampled at discrete times from the trajectories of three-time continuous Gaussian processes, $(X_t^A)_{t\geq 0}$, $(X_t^B)_{t\geq 0}$ and $(X_t^C)_{t\geq 0}$. The three processes share the same covariance function K given as

$$K(s,t) = \sigma \cdot \exp\left(-\frac{|s-t|}{l}\right),$$

where $K(s,t) = Cov(X_s^A, X_t^A) = Cov(X_s^B, X_t^B) = Cov(X_s^C, X_t^C)$. The mean of the processes is given by the function m

$$m(t,\mu) = \mu \cdot (t - t_0)^2 + c,$$

with three different choices of the parameter μ, namely $\mathbb{E}(X_t^A) = m(t,\mu^A)$, $\mathbb{E}(X_t^B) = m(t,\mu^B)$ and $\mathbb{E}(X_t^C) = m(t,\mu^C)$. In particular, the parameters have been set to the values $\mu^A = 6$, $\mu^B = 0$, and $\mu^C = -6$. The dataset consists of $n = 50$ curves for each process, hence $N = 150$ sampled curves altogether, and the parameters shared by the three processes are $\sigma = 10^4$, $l = 1, t_0 = 10$ and $c = 10^2$. The time points at which the trajectories are sampled are randomly chosen, both their number and their position. For each trajectory, the number of sampling time points is given as the $\max(3,r)$, where r ~Binomial $(7, 0.5)$, and uniformly distributed in the time interval $[1, 22]$. The dataset is illustrated in Fig. S10-panel A and colored by label: A for X^A, B for X^B, and C for X^C.

Setting $\boldsymbol{p}$. As the dataset contains the true labels (A, B, and C), we can evaluate the performance of CONNECTOR as a classifier, by setting $G = 3$ and by finding a correspondence between the CONNECTOR clusters and the groups of identically labeled curves, that is the group of sampled curves

from X_t^A, from X_t^B and from X_t^C. The point in this experiment is the choice of p, the dimension of the spline basis. This parameter defines the flexibility of the functional clustering model (2), larger values of p corresponding to higher flexibility, hence smaller bias and larger variance. Good values for this parameter are expected to be central, with a preference for small values in order to reduce the number of parameters to be estimated. The cross-loglikelihood plot, see Fig. S9, suggests choosing $p = 4$. This choice is motivated by the compromise between a large log-likelihood and a small number of parameters to be estimated. This choice is furthermore supported by the fDB index, see eq. (5), and the total tightness T, see eq. (4), calculated as p varies, for $G = 3$ and for the most frequent output clustering, see Fig. S11-panels A and C. The value $p = 4$ corresponds to the minimum fDB and to an elbow in the total tightness plot. The stability of the clustering is reported in Fig. S11-panel B and for $p = 4$ is large enough. The clustered curves are illustrated in Fig. S10-panel B and colored by the true label. The examination of the plots of the curves in the CONNECTOR clusters leads to a reasonable link between the CONNECTOR cluster 1 with the label A, the CONNECTOR cluster 2 with the label C, and the CONNECTOR cluster 3 with the label B.

The performance of CONNECTOR in clustering together sampled curves from trajectories of processes with the same label is evaluated by plotting the True Positive (TP) rate and the False Positive (FP) rate, for each label, in Fig. S11-panels D, E and F. The value $p = 4$ is the only value at which, for all three labels, TP rate is large and FP rate is small. Notice that the label B is the label that is most often confused. This is not surprising as the process X_t^B has zero mean, hence its trajectories are driven by the variance. With a small number of sampling points, a small positive fluctuation can be wrongly classified in CONNECTOR cluster 1, while a small negative fluctuation can be wrongly classified in CONNECTOR cluster 2. The best clustering reported in Fig. S10-panel B shows that indeed only one curve sampled from C is wrongly assigned to CONNECTOR cluster 3 and some curved sampled from B are wrongly assigned to CONNECTOR clusters 1 and 2. Though, it is important to emphasize that all the wrong assignments are actually very well fitting in the designated CONNECTOR cluster.

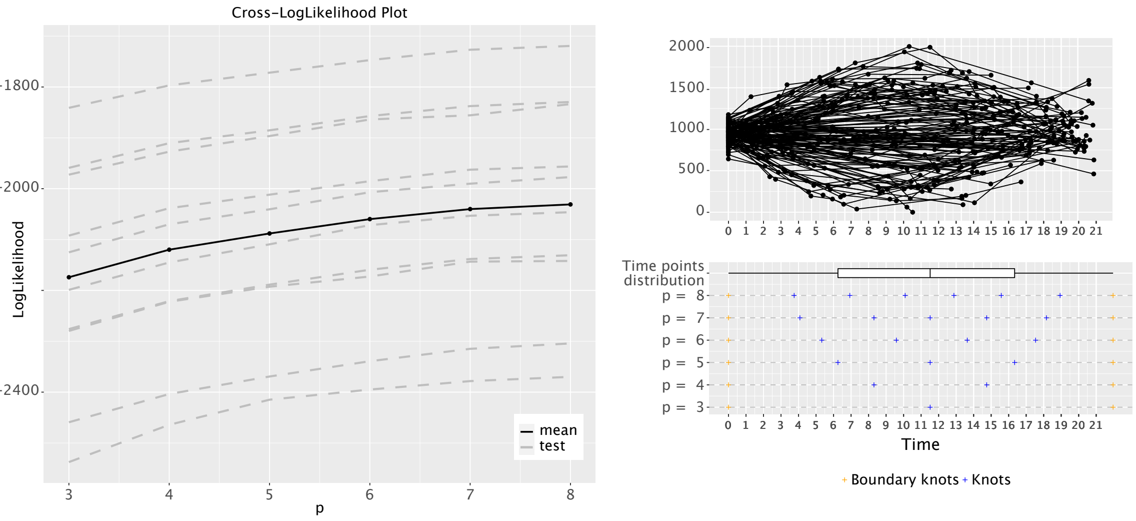

Fig. S9: **The simulated dataset.** The cross-loglikelihood plot on the left panel. The solid black line is the mean loglikelihood, and the dashed grey lines are the 10-fold cross-loglikelihoods.

Setting G. In this second experiment, we show the performance of the proposed method for choosing G, that is the analysis of the fDB violin plots, the Tightness violin plots, and the stability matrices. In Fig.S12 the plots are reported, for the suggested choice of $p = 4$ and for $G = 2, 3, 4$. The values of the fDB indices are very good, the distributions of the indices on different runs lie entirely below the constant line at 0.6 both for the choices $G = 2$ and $G = 3$ (good fDB values are considered to be smaller than 1). The case $G = 4$ suffers from a higher instability, as shown by the dots around the values 0.65 and 1.2 and by the stability matrices. Hence, we would suggest $G = 3$ be the optimal number of clusters.

S7.2 Robustness for the spline basis dimension choice

In this Section we give some additional details on the performances of CONNECTOR for different choices of the spline basis dimension parameter p, hence when setting p as suggested by CONNECTOR and when setting p to other values.

To explore this issue, we run CONNECTOR on the PDX growth curves analyzed in Section 3, for different choices of the parameter p. For all the values $p = 3, 4, 5, 6, 7, 8$, the fDB, tightness, and stability indices suggest choosing $G = 4$, similarly to the case $p = 4$ reported in Fig. S2. Hence in Fig. S13 all curves are plotted with $G = 4$.

The cross-loglikelihood plot in Fig. S13 suggests choosing $p = 4$, as it is the value at which the cross-loglikelihood increases to a measure that is maintained steadily up to $p = 8$, and $p = 4$ is small enough to be conservative with the number of parameters to estimate and to keep the model flexible but not overfitting. The fDB plot shows that the best clusterings are obtained for $p = 3$ and $p = 4$, at which the fDB is smaller. The stability and the tightness values confirm that the best choices are for $p = 3$ and $p = 4$.

As the parameter p determines the flexibility of the model, smaller values are associated with models that show larger bias and smaller variance, while larger values are associated with models that better fit the points of the curves, but variance increases. This can be appreciated in Fig. S14, where the cluster centers (the mean curves for each cluster) are plotted for three values of $p = 4, 5, 6$. As p increases, it is evident that the estimated mean

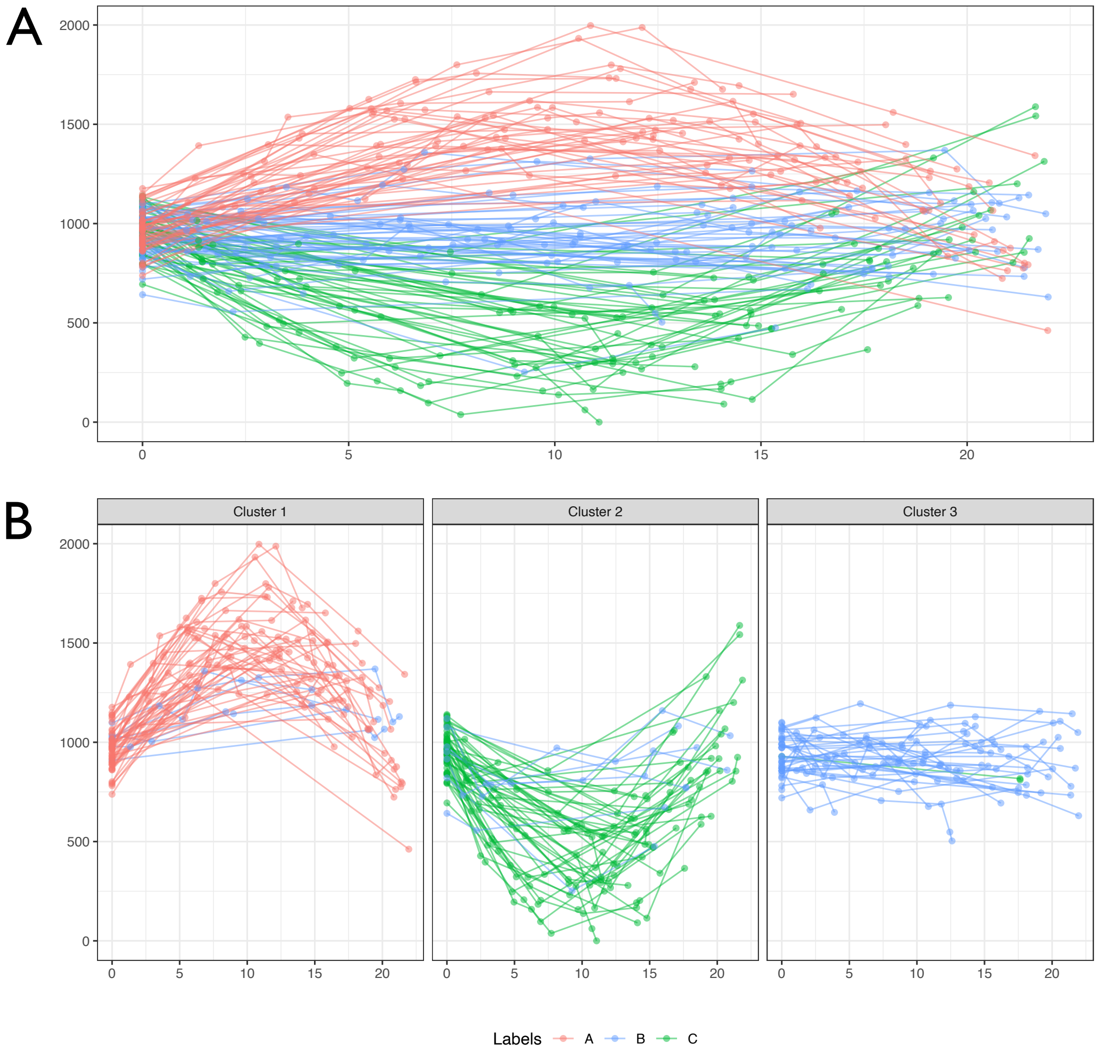

Fig. S10: **The simulated dataset. Panel A:** the complete simulated dataset colored by true label. Red curves are simulated from process X_t^A, green curves are simulated from process X_t^C, and blue curves are simulated from process X_t^B. **Panel B:** The CONNECTOR clustering of the simulated dataset.

curves exhibit more fluctuations which are the result of a tighter fitting to the data points, but the resulting estimation of the curves and hence of the clustering are deteriorated (as indicated by the resulting indices).

However, we should notice that CONNECTOR is very robust to choices of the parameter p which are less than optimal. Indeed up to $p = 5$ the indices of the resulting clusterings are all very good.

S7.3 Robustness for decreasing numbers of sampled curves and sampling times.

In this Section, we tackle the performance of CONNECTOR when the dataset is downsampled. There are two cases that lead to a poor dataset in this framework: the first one is that the number of sampled curves is small, the second one is that the curves are sampled at few time points.

The experiment setup is designed as follows: we start from the PDX growth curves dataset analyzed in Section 3 and we perform two degradation steps, each of them deleting the 25% of the sampling time points randomly. After these two steps, we have three datasets: the original dataset, the dataset reduced to a proportion equal approximately to the 75% of the original sampling time points and the dataset reduced to a proportion equal approximately to the 50% of the original sampling time points. The characteristics of the three datasets are illustrated in Fig. S15. The number of sampling time points is actually reduced, see Fig. S15-panel A, and so is the number of curves as well, S15-panel B. Indeed, we choose to run CONNECTOR on the curves with a minimum length equal to three, i. e. with at least three sampling time points, hence a reduction of the number of sampling time points leads to a reduction of the number of curves as well. However, the distribution of the number of sampling time points per curve is similar in the three datasets, see S15-panel C, being around the value 4, with few exceptions.

The indices of the performance of CONNECTOR on the three datasets are plotted in Fig. S15-panels D-E-F and show that the overall goodness of the final clustering decreases, consistently for the three indices fDB, stability, and tightness, as the original dataset degrades. Hence the three indices

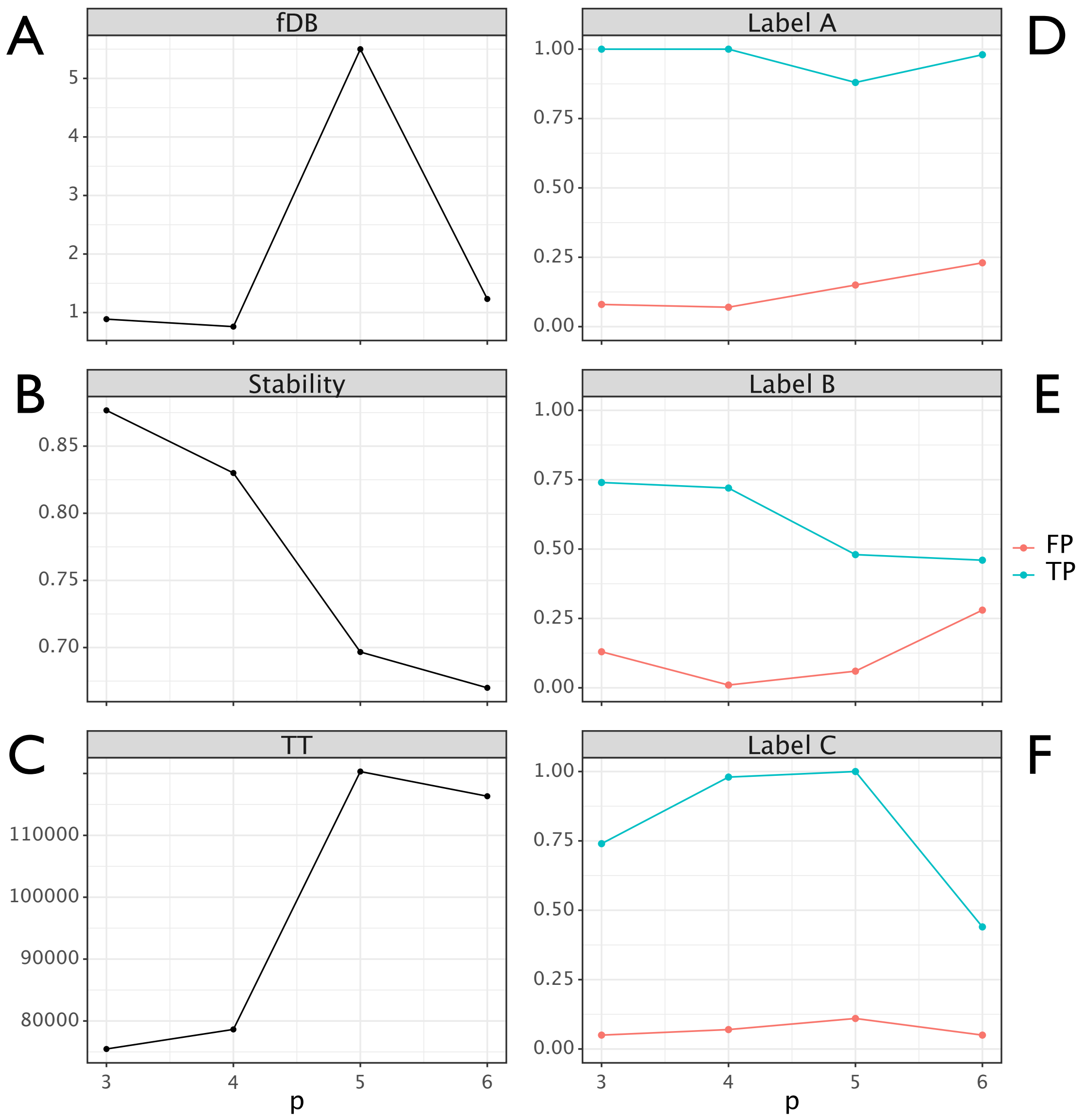

Fig. S11: **The simulated dataset.** The fDB (panel A), Total Tightness (panel C), and Stability (panel B) as p varies. False Positive rate (FP) and True Positive rate (TP) as p vary and for the three true labels, label A (panel D), label B (panel E) and label C (panel F).

are able to highlight the worsening of the datasets. However, the punctual values of the three indices are very good for all three datasets, suggesting that CONNECTOR is very robust to the downsampling. This is confirmed by the cluster centers, plotted in Fig. S16, that are very similar in the final clustering on the three different datasets.

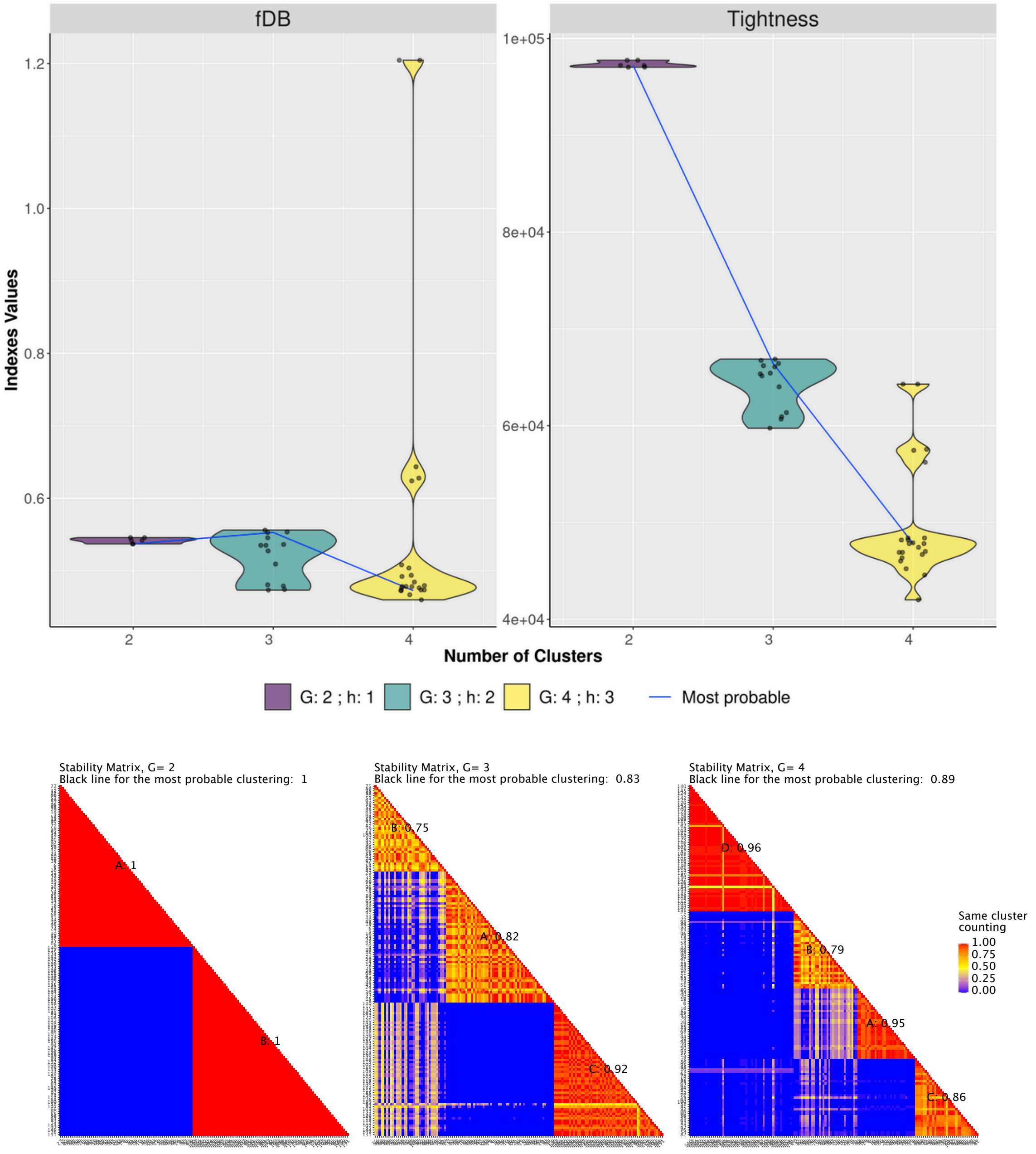

Fig. S12: **The simulated dataset.** Violin plots of the fDB and of the Total Tightness (first row). Stability matrices for $G = 2, 3, 4$ (last row).

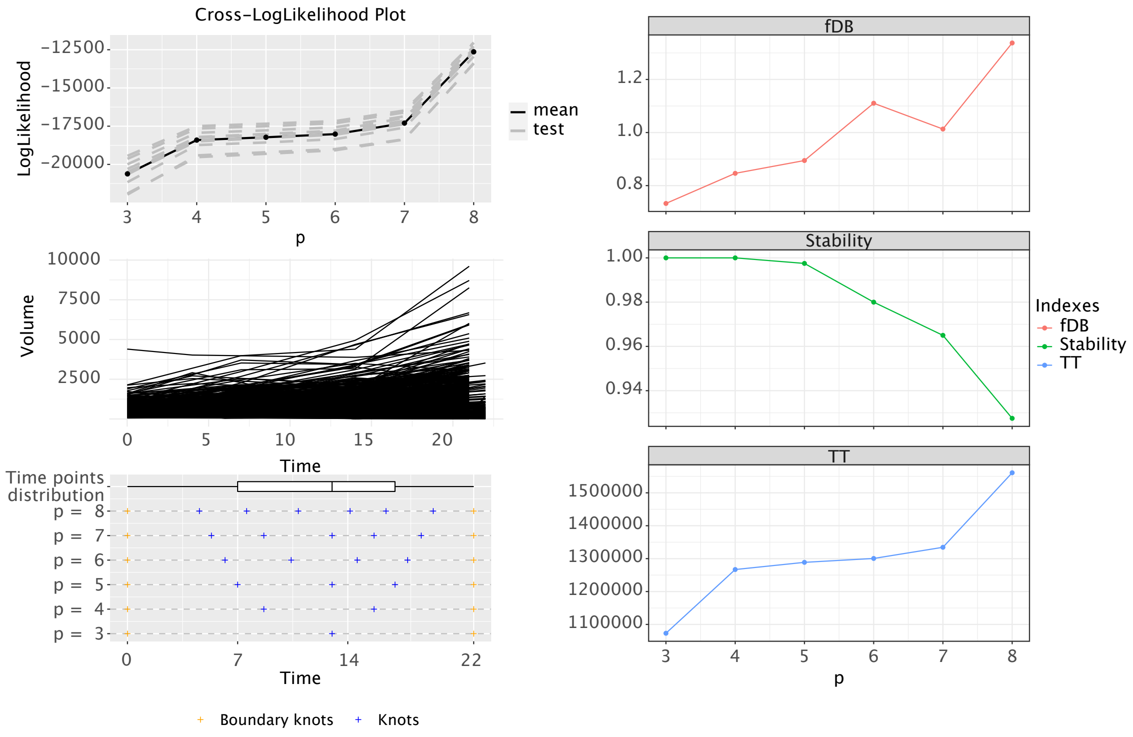

Fig. S13: **The PDX dataset.** Cross-loglikelihood plot, sample curves, and knots positions as p varies (left column). fDB, Stability and Total Tightness as p varies (right column).

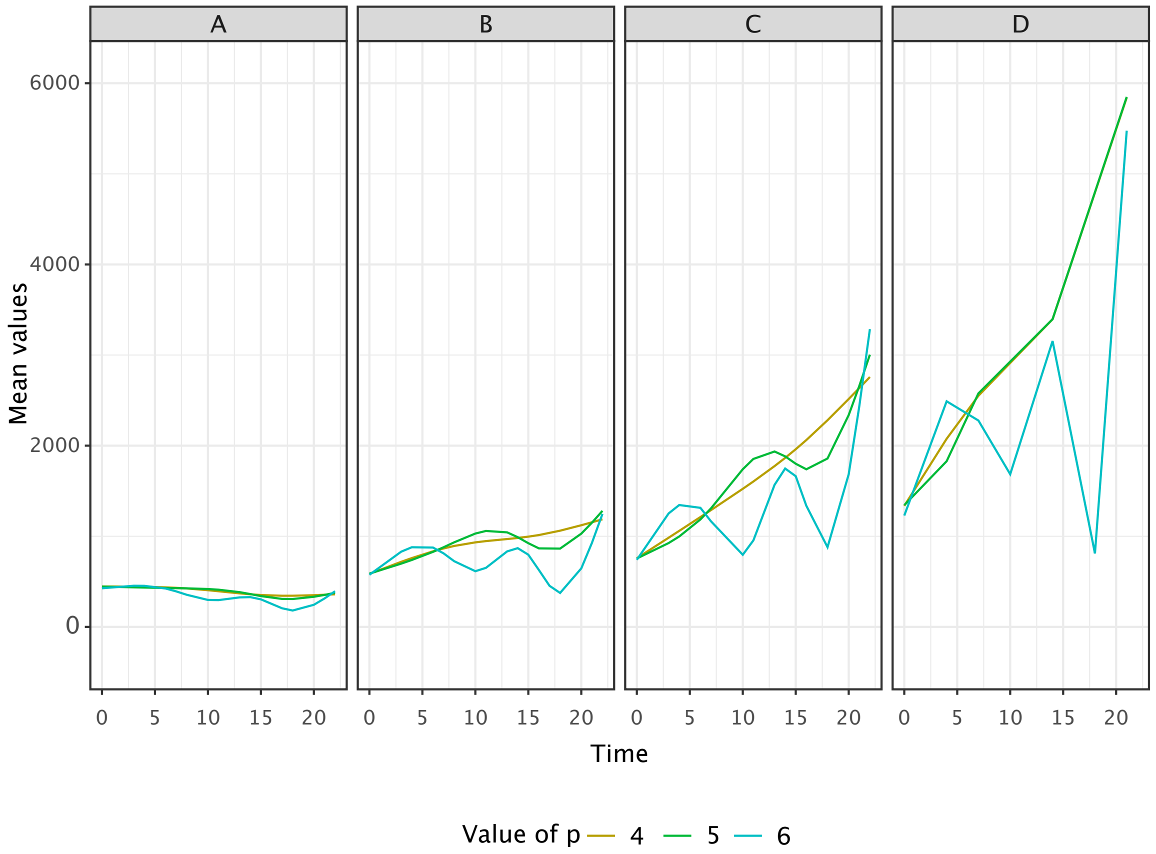

Fig. S14: **The PDX dataset.** Cluster centers for three different choices of $p = 4, 5, 6$.

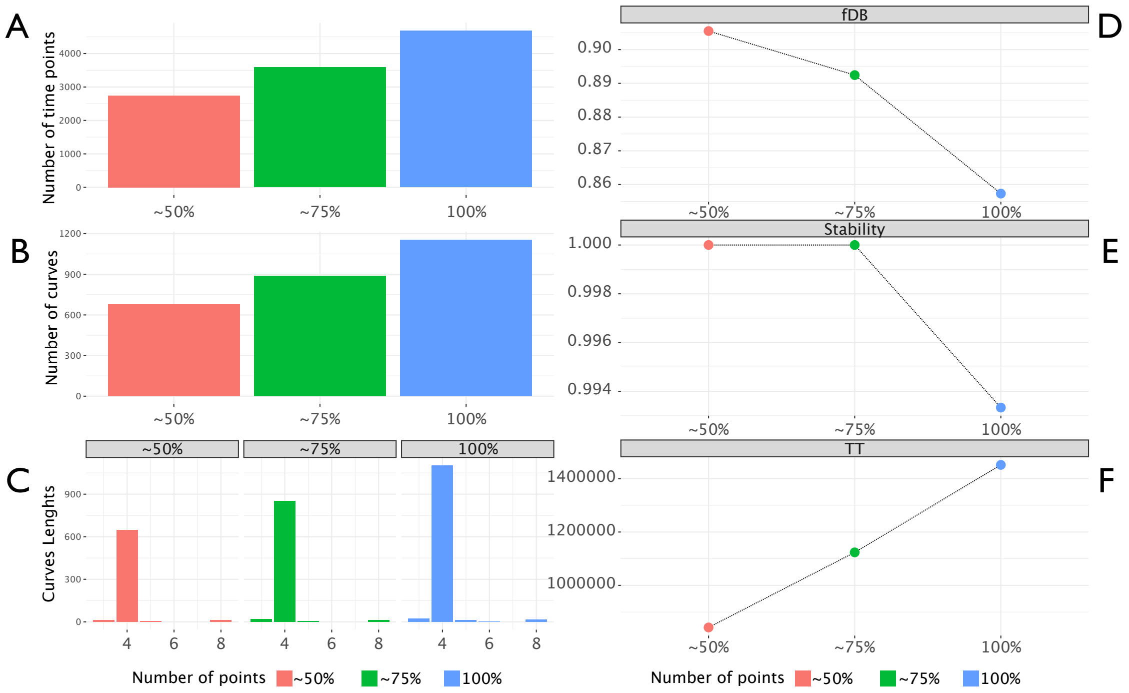

Fig. S15: **The reduced PDX datasets.** Total number of sampling time points (panel A), number of curves (panel B), and distribution of the curve lengths (panel C). fDB (panel D), Stability (panel E), and Total Tightness (panel F) for the clusterings of the three datasets: the complete (blue), the reduced to approximately 75% of the number of points (green), and the reduced to approximately the 50% of the number of points (red).

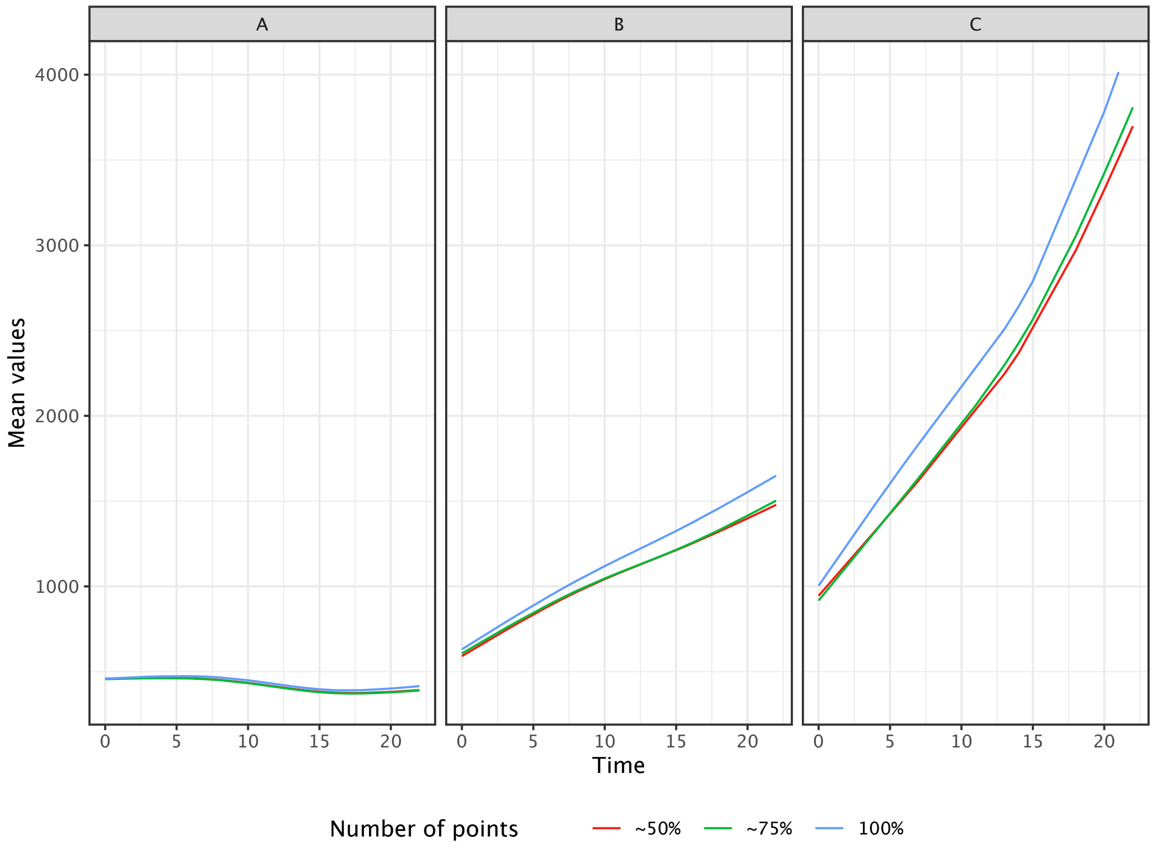

Fig. S16: **The reduced PDX datasets.** Cluster centers for three datasets: the complete (blue), the reduced to approximately 75% of the number of points (green), and the reduced to approximately 50% of the number of points (red).

S8 Performance of different clustering methods on sparse and irregularly sampled curves

In this Section, we review the performances of different clustering procedures that could be implemented on longitudinal data.

Clustering with Classical Growth Models. As we address growth curves in this paper, we cannot skip mentioning the classical growth models that have been introduced in the literature. In the following, we review the main growth models (Malthus, Gompertz, and logistic) and the performance of a clustering method based on the direct estimation of the parameters involved. Indeed, each sampled curve could be represented as the array of the estimated values of the parameters of the fitted growth model. In doing so, the full sample of curves becomes a sample of points in $\mathbb{R}^n$, where n is the number of parameters of the growth model, and the clustering procedure can be performed using classical clustering methods for data points.

Let us briefly review the most popular growth models. These models are usually formulated in terms of ordinary differential equations (ODEs) that relate the growth rate of the tumor to its current state and range from the simple one-parameter exponential growth model to more advanced models that contain more parameters.

The *Malthus model* was one of the first mathematical models introduced to study and model exponential-linear population growth. It assumes that every cell continuously passes through the cell cycle giving birth to two daughter cells at regular intervals. Then, the number of cancer cells and therefore the volume of the tumor would increase exponentially over time. In particular, the Malthus model is described by the following ODE:

$$\begin{cases} \dfrac{dV(t)}{dt} & = \alpha V(t) \\ V(t_0) & = V_0, \end{cases} \tag{S15}$$

where $V(t)$ is the total tumour volume at time t, α is a constant for the growth rate, and V_0 is the tumour volume at the initial time t_0.

Successively, the *Gompertz model* was derived from a generalisation of the Malthus model, assuming that the relative growth rate is not constant, but time-dependent with a decreasing behavior at constant positive rate β:

$$\begin{cases} \dfrac{dV(t)}{dt} & = \alpha(t)V(t) \\ \dfrac{d\alpha(t)}{dt} & = \beta\alpha(t) \\ V(t_0) & = V_0 \\ \alpha(t_0) & = \alpha_0. \end{cases} \tag{S16}$$

Finally, in the *logistic model*, the relative growth rate decreases linearly, when the population size increases, according with the assumption that resources are limited. Thus, the ODEs defining the model are:

$$\begin{cases} \dfrac{dV(t)}{dt} & = \alpha V(t) \left(\frac{K-V(t)}{K}\right) \\ V(t_0) & = V_0, \end{cases} \tag{S17}$$

where α is a coefficient related to population kinetic and K is the so-called carrying capacity, which represents the maximum population size a particular environment can support.

For each model above reported, a clustering algorithm can be implemented in a two steps procedure:

1. For every i, estimate the model parameters by fitting the i-th sampled curve. The result is an array $(p_{i_1}, \ldots, p_{i_n})$, where n is the number of model parameters. Each sampled curve is now represented as a data point in dimension n.
2. Cluster the full sample of data points $(p_{i_1}, \ldots, p_{i_n})_{i=1}^N$ by a classical clustering algorithm. We chose a k-means algorithm.

The procedure has been tested and the performance is reported here.

The comparison setup. We tested the clustering with classical growth models on four sets of growth curves, see Figure S17. The four CONNECTOR analyses are reported at `https://qbioturin.github.io/connector/examples/`.

For each sample, we first fitted the Malthus, Gompertz and logistic models. The estimated parameters for each curve in each of the four samples are reported in Tables S4, S5, S6 and S7.

The data points are then clustered through a k-means clustering algorithm. The optimal number of clusters is determined by evaluating the *Total Tightness*, see eq. (4) (in the main paper), and the *fDB indexes*, see eq. (5) (in the main paper), calculated using the distance between curves, see eq. (3) (in the main paper). The quality of the final clustering of the corresponding curves is evaluated by the fDB index. The results are reported in Table S8. Those values will be compared to the values of the same index on clustering of the same samples through functional methods, discussed in the following paragraph. As a first comment, notice that the classical models are not flexible enough to fit the curves in test 4, which exhibit more complex dynamics than a "simple" growth.

Review and Comparison of Functional Clustering Methods. Clustering functional data is generally a difficult task because of the infinite dimensional space from which data are sampled. Different approaches have been proposed along the years reviewed in Müller (2005) and Jacques and Preda (2014).

The most popular approach consists of reducing the problem to a finite dimensional setting by approximating data with elements from some finite dimensional space. Afterwards, clustering algorithms for finite dimensional data can be run. The reducing dimension step, often denoted as *filtering* step, consists in approximating the curves into a finite basis of functions. Spline basis is one of the most common choice because of their optimal properties. Another dimension reduction technique is the functional principal component analysis, based on the Karhunen-Loeve expansion of a square integrable L^2 stochastic process. The implementation of functional principal components also requires some form of regularisation, which can be achieved with smoothing methods, see Ramsay and Silverman (2005).

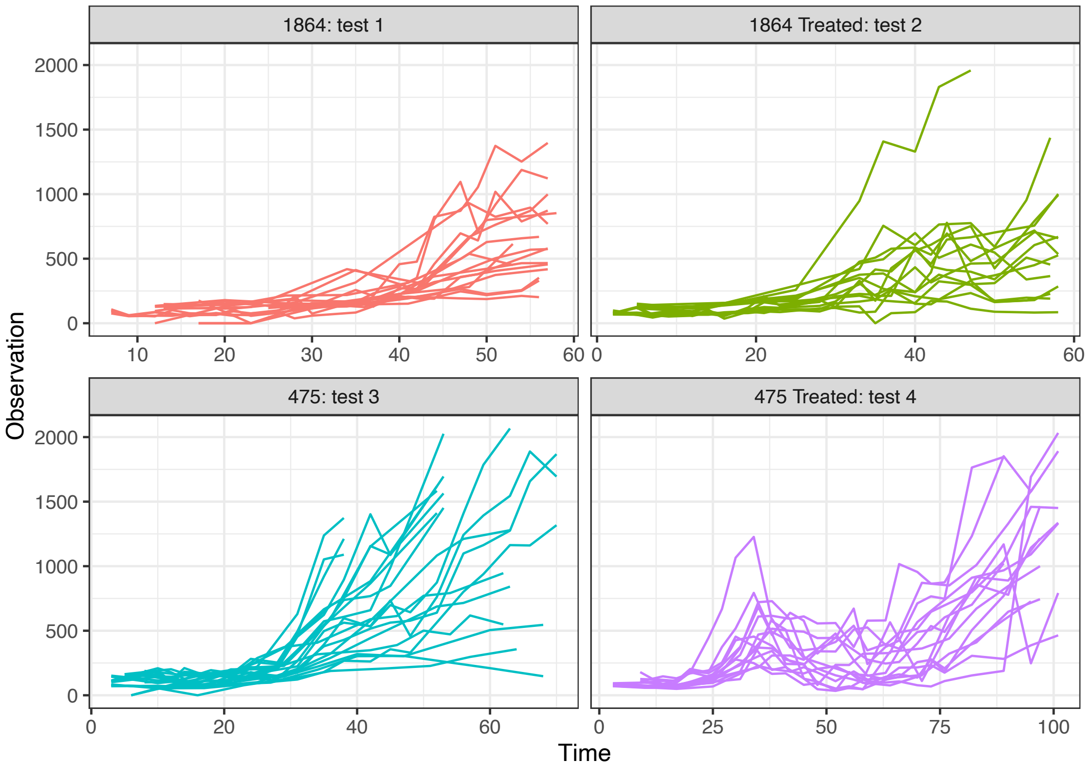

Fig. S17: The four tests samples.

Table S4. Estimated parameters for each sampled curve in Test 1.

Malthus		Logistic			Gompertz		
V_0	α	V_0	K	α	V_0	α_0	β
68.78308	0.04445	39.39801	1701.15668	0.06589	31.56854	0.08716	0.01548
36.89524	0.04989	6.40114	656.23237	0.10991	1.83531	0.24387	0.03739
11.61218	0.07304	11.55314	99999.99076	0.07323	11.61144	0.07304	0
28.55079	0.05322	15.54528	1221.30806	0.07456	17.80942	0.07694	0.00828
22.0811	0.05481	1.35357	499.86858	0.14483	0.00012	1.08476	0.07014
20.43031	0.0543	4.40454	531.67804	0.10741	0.08896	0.44936	0.05004
40.12871	0.0477	39.95205	99999.91164	0.04787	40.12695	0.0477	0
91.71518	0.0414	0.62068	889.00398	0.19828	0	1.85772	0.0893
47.13489	0.05348	46.75451	99999.80804	0.05378	47.13253	0.05348	0
125.2202	0.00929	84.84368	210.67975	0.06516	78.17013	0.05303	0.05259
90.93223	0.01975	90.91487	99999.99218	0.01979	90.93134	0.01975	0
46.32061	0.04917	20.80527	1135.41884	0.07941	11.54091	0.13244	0.02368
65.85038	0.02732	65.81126	99999.8859	0.02737	65.84926	0.02732	0
25.90587	0.06918	0.0866	1198.44381	0.21987	0	1.36521	0.06464
18.81232	0.0585	13.72665	1920.26111	0.06913	18.81129	0.0585	0
27.59306	0.07141	0.51379	1598.25243	0.17844	0	1.39001	0.06416
22.62525	0.06707	0.17156	1014.09897	0.19457	2e-05	1.10764	0.05973

On the other hand, nonparametric methods for clustering have been proposed, see Ferraty and Vieu (2006a). They consist generally in defining specific distances or dissimilarities for functional data and then apply clustering algorithms as hierarchical clustering or k-means.

Finally, model-based clustering techniques have been developed. In this cases the observations are modeled as mixture distributions. Two main currents have been explored: one which models the functional principal components scores Bouveyron and Jacques (2011) and another one which models directly the expansion coefficients in a finite basis of functions James and Sugar (2003).

The advantages and disadvantages of each method are summarized in Jacques and Preda (2014). We do not enter the discussion here and refer the reader to the original paper. As the conclusion of the critical analysis presented in Jacques and Preda (2014) is that model-based techniques are better

Table S5. Estimated parameters for each sampled curve in Test 2.

Malthus		Logistic			Gompertz		
V_0	α	V_0	K	α	V_0	α_0	β
107.86528	0.04256	100	99999.99998	0.04422	100	0.04511	0.00093
148.82615	0.00778	100	224.47694	0.07737	100	0.0509	0.06217
115.84188	0.06204	4.82149	2131.27134	0.17775	0	2	0.0936
128.61429	0.02246	100	630.47178	0.04402	95.00862	0.0449	0.01968
171.50539	0.00511	60.1021	225.35314	0.1638	48.06176	0.21451	0.13895
223.56588	0.01579	0.01163	508.82563	0.40541	8.27013	0.38886	0.09368
112.0361	0.03331	6.97696	636.43582	0.15673	0.0052	1.15334	0.09802
59.8971	0.04839	21.37189	1176.66403	0.08888	13.74335	0.14067	0.02602
58.27364	0.04228	35.822	1016.18853	0.06552	31.05498	0.08178	0.01681
78.61606	0.00571	71.6155	111.95701	0.04039	71.78805	0.01499	0.03233
54.98902	0.04078	9.30408	563.20968	0.11093	7.13232	0.1841	0.03788
51.0954	0.04567	41.09212	2140.78544	0.05549	40.61632	0.05864	0.00615
60.65665	0.04868	19.04046	1142.30986	0.09399	6.62424	0.19625	0.03419
56.36788	0.02682	50.57229	642.90686	0.0357	51.84238	0.03257	0.0054

Table S6. Estimated parameters for each sampled curve in Test 3.

Malthus		Logistic			Gompertz		
V_0	α	V_0	K	α	V_0	α_0	β
42.70428	0.0337	22.53575	465.28195	0.06276	12.30043	0.11177	0.0279
72.6787	0.03076	36.66447	702.80793	0.06164	24.64689	0.09753	0.02596
94.64953	0.01412	4.20258	237.95861	0.16797	0.14039	0.81406	0.10959
125.83268	0.02544	100	1127.65656	0.03918	100	0.03856	0.00993
122.30407	0.03426	66.98609	1261.45671	0.06391	51.28875	0.0903	0.02379
64.88787	0.06204	43.44689	4531.19706	0.07783	36.04188	0.0955	0.01074
58.08083	0.06463	1.83068	1762.01364	0.17582	0	1.61343	0.07838
85.06345	0.03739	52.49335	1567.833	0.05678	59.37865	0.05581	0.00881
56.98349	0.06218	22.27463	2412.11609	0.09673	18.13171	0.12947	0.01806
76.23054	0.04685	3.95032	1428.83744	0.13278	1e-05	1.30867	0.06983
73.84691	0.05908	15.66189	1961.0405	0.11656	2.37164	0.30248	0.04128
17.98485	0.11056	17.6945	99999.9983	0.11128	17.98388	0.11056	0
49.92763	0.06386	34.97924	4478.20046	0.07744	36.29659	0.08126	0.00594
25.63826	0.08205	25.00302	99999.99928	0.08288	25.63639	0.08205	0
80.72953	0.05891	13.29765	2039.99107	0.12414	2.02834	0.31975	0.04238
20.68873	0.10578	20.35612	99999.9974	0.1065	20.68765	0.10578	0
33.16078	0.0661	31.79952	65601.37522	0.06725	33.15802	0.06611	0
35.56968	0.05302	5.0395	1784.69542	0.09982	0.19676	0.31705	0.03201
55.18988	0.05078	38.27897	6156.91077	0.06079	27.81077	0.07912	0.00816
84.37937	0.04521	27.16628	2799.89852	0.07617	10.51689	0.14452	0.02174
17.66668	0.11646	0.17484	1686.25935	0.28453	0	1.85498	0.08425

performing, we decided to rely on such techniques. In particular on the two methods presented in James and Sugar (2003) and in Bouveyron and Jacques (2011). We tested both techniques on the “general growth curve" type of data which are the object of this paper.

The comparison setup. The two selected clustering procedures are both implemented in a software. The clustering procedure introduced in Bouveyron and Jacques (2011) is implemented in the **R** package ‘funHDDC’, maintained and available for download from CRAN. The method by James and Sugar (2003) is implemented in an **R** function ‘fclust’ available directly from James’s webpage *http://faculty.marshall.usc.edu/gareth-james/index.html*.

We tested both methods on four sets of growth curves from Figure S17, the same dataset on which the clustering with classical growth models have been tested, see Table S8. The results are summarised in Table S9. Notice that ‘funHDDC’ includes a model selection function to help the user set the optimal number of clusters, while ‘fclust’ has been integrated with the model selection procedures introduced in CONNECTOR. Moreover, the functional latent mixture model presented in Bouveyron and Jacques (2011) can be reduced to different submodels by constraining model parameters within or between groups. Here we tested the submodels which are denoted as $[a_{kj}, b_k, Q_k, d_k]$, $[a_k, b_k, Q_k, d_k]$, $[a_{kj}, b, Q_k, d_k]$ and $[a_k, b, Q_k, d_k]$.

Notice that both fclust and funHDDC result in very good fDB indexes, compared to the fDB indexes reported in Table S8 for the classical models. Both the functional clustering methods are based on very flexible models, that can adapt to data. On the contrary, the simple classical models have too few degrees of freedom and cannot adjust to the sampled curves.

As shown in Table S9, the method by James and Sugar (2003) lead to far better results. The conclusion of the comparison integrates the study performed in Jacques and Preda (2014), as here, strongly sparse and irregularly sampled curves are considered, few points per curve $\sim$ 7 to 20 instead of $\sim$ 31 to 241.

Table S7. Estimated parameters for each sampled curve in Test 4.

Malthus		Logistic			Gompertz		
V_0	α	V_0	K	α	V_0	α_0	β
112.97695	0.02399	100	12992.23318	0.0263	100	0.02764	0.00198
82.27909	0.02809	81.90911	99999.90899	0.02824	73.62575	0.03213	0.00217
111.74149	0.01612	100	1e+05	0.01773	0.00029	2	0.14378
123.2589	0.00839	15.10299	213.24741	0.16083	20.24044	0.22409	0.09495
229.54567	0	1e-05	269.55768	0.67233	0.00372	2	0.17934
36.69513	0.02804	36.68232	99999.99282	0.02808	36.69373	0.02804	0
54.51746	0.03156	54.30083	99999.98986	0.03169	54.51437	0.03156	0
121.71725	0.02903	100	99999.99998	0.03176	100	0.03288	0.001
46.1963	0.04112	45.50316	99999.99969	0.04146	46.19206	0.04112	0
141.07528	0.0202	100	1e+05	0.02497	100	0.0249	0
43.83091	0.03301	43.64035	99999.99931	0.03313	43.82805	0.03301	0
127.11997	0.01784	100	99999.99999	0.02127	100	0.02122	0
62.25641	0.03366	61.77783	99999.99054	0.03387	62.25232	0.03366	0
37.65773	0.02855	37.65595	99999.9931	0.02858	37.65623	0.02855	0
297.33316	0.00647	0.00024	471.42615	0.71313	0.00734	2	0.18113

Table S8. Test results for the classical models.

	**Malthus**		**Gompertz**		**logistic**	
	**Number of clusters**	**fDB index**	**Number of clusters**	**fDB index**	**Number of clusters**	**fDB index**
test 1	$G = 4$	3.753	$G = 4$	1.9956	$G = 5$	4.8957
test 2	$G = 5$	0.8628	$G = 6$	2.1597	$G = 5$	1.3979
test 3	$G = 5$	1.631	$G = 4$	0.8986	$G = 6$	0.7092
test 4	$G = 6$	1.7203	$G = 3$	2.0086	$G = 4$	2.0892

Table S9. Test results from fclust and funHDDC.

	**fclust**			**funHDDC**		
	**Parameters**	**Number of clusters**	**fDB index**	**Best Model Parameters**	**Number of clusters**	**fDB index**
test 1	$p = 3$<br>$h = 1$	$G = 4$	0.4831	$[a_k, b, Q_k, d_k]$<br>$p = 3$	$G = 2$	0.7932
test 2	$p = 3$<br>$h = 1$	$G = 4$	0.4594	$[a_k, b, Q_k, d_k]$<br>$p = 4$	$G = 3$	0.8853
test 3	$p = 3$<br>$h = 1$	$G = 4$	0.3897	$[a_k, b, Q_k, d_k]$<br>$p = 4$	$G = 3$	0.5539
test 4	$p = 3$<br>$h = 2$	$G = 4$	0.4049	$[a_k, b, Q_k, d_k]$<br>$p = 3$	$G = 3$	1.4478

Supplementary References

(2022). Ebi quick go. `https://www.ebi.ac.uk/QuickGO/annotations?goUsage=descendants&goUsageRelationships=is_a,part_of,occurs_in&goId=GO:0031424`.

Bouveyron, C. and Jacques, J. (2011). Model-based clustering of time series in group-specific functional subspaces. *Adv. Data Anal. Classif.*, **5**(4), 281–300.

Castellarin, M. *et al.* (2013). Clonal evolution of high-grade serous ovarian carcinoma from primary to recurrent disease. *Journal Pathology*, **229**, 515–524.

Erriquez, J. *et al.* (2016). Xenopatients show the need for precision medicine approach to chemotherapy in ovarian cancer. *Oncotarget*, **7**(18), 26181–26191.

Ferraty, F. and Vieu, P. (2006a). *Nonparametric functional data analysis*. Springer Series in Statistics. Springer, New York. Theory and practice.

Ferraty, F. and Vieu, P. (2006b). *Nonparametric functional data analysis: theory and practice*. Springer Science & Business Media.

Hothorn, T. (2017). *Maximally selected rank statistics with several p-value approximations*.

Jacques, J. and Preda, C. (2014). Functional data clustering: a survey. *Advances in Data Analysis and Classification*, **8**(3), 231–255.

James, G. M. and Sugar, C. A. (2003). Clustering for sparsely sampled functional data. *Journal of the American Statistical Association*, **98**(462), 397–408.

Kassambara, A. (2021). *Drawing Survival Curves using 'ggplot2'*.

Love, M. I. *et al.* (2014). Moderated estimation of fold change and dispersion for RNA-seq data with DESeq2. *Genome Biology*, **15**(12), 550.

McPherson, A. *et al.* (2016). Divergent modes of clonal spread and intraperitoneal mixing in high-grade serous ovarian cancer. *Nature genetics*, **48**(7), 758–767.

Müller, H.-G. (2005). Functional modelling and classification of longitudinal data. *Scand. J. Statist.*, **32**(2), 223–246. With discussions by Ivar Heuch, Rima Izem, and James O. Ramsay and a rejoinder by the author.

Ramsay, J. O. and Silverman, B. W. (2005). *Functional data analysis*. Springer Series in Statistics. Springer, New York, second edition.

RStudio, Inc (2014). *shiny: Easy web applications in R*. url: `http://shiny.rstudio.com`.

Schmitt, M. W. *et al.* (2016). The influence of subclonal resistance mutations on targeted cancer therapy. *Nature Reviews. Clinical Oncology*, **13**(6), 335–347.

Schwarz, R. *et al.* (2015). Divergent modes of clonal spread and intraperitoneal mixing in high-grade serous ovarian cancer. *PLoS Medicine*, **12**(2).

Shannon, C. E. (1948). A mathematical theory of communication. *The Bell system technical journal*, **27**(3), 379–423.

Therneau, T. M. (2022). *A Package for Survival Analysis in R*. R package version 3.3-1.

Van der Maaten, L. and Hinton, G. (2008). Visualizing data using t-sne. *Journal of machine learning research*, **9**(11).

Wu, T. *et al.* (2021). clusterProfiler 4.0: A universal enrichment tool for interpreting omics data. *The Innovation*, **2**(3), 100141.

Yu, G. *et al.* (2012). clusterProfiler: an R Package for Comparing Biological Themes Among Gene Clusters. *OMICS: A Journal of Integrative Biology*, **16**(5), 284–287.
